# *Rhizobium*-Enhanced Drought Tolerance in Red Kidney Beans Through Modification of Transcriptome and Microbial Communities

**DOI:** 10.3390/microorganisms13092153

**Published:** 2025-09-16

**Authors:** Xiaoliang Li, Chunguo Huang, Qian You, Gaiya Jia, Yongjunlin Tan, Shenjie Wu, Zhaosheng Kong, Lixiang Wang

**Affiliations:** Shanxi HouJi Laboratory, College of Agriculture, Shanxi Agricultural University, Taiyuan 030031, China

**Keywords:** drought stress, microbiome community, red kidney beans, *Rhizobium* PV-6, transcriptome

## Abstract

Drought is a significant abiotic stressor affecting crops globally. Beneficial microorganisms, such as rhizobia, have been shown to enhance crop resilience to such stresses. In this study, we isolated a highly efficient rhizobacterial strain (*Rhizobium* sp. PV-6) from the root system of *Phaseolus vulgaris* and systematically investigated the phenotypic and physiological responses of the plants across seven growth stages under four treatments: W-NO (watering without inoculation of *rhizobium*), W-RHI (watering with inoculation of *rhizobium*), D-NO (drought without inoculation of *rhizobium*), and D-RHI (drought with inoculation of *rhizobium*). We also examined the variation in microbial communities in rhizosphere and root compartments. Physiological analyses revealed that *rhizobium* inoculation significantly enhanced plant height, fresh weight and dry weight, root length, lateral root number, and nodule number of red kidney beans. Alpha diversity analysis suggested that the microbial communities in the roots and rhizosphere of red kidney beans show different variant distributions. Beta diversity and species difference analysis revealed that drought treatments (D-NO, D-RHI) recruit *Shinella*, *Nocardioides*, *Agromyces*, *Pseudomonas*, and *Ensifer* at rhizosphere compartments, while D-RHI enrich *Pseudomonas*, *Sphingobacterium*, *Paenibacillus*, *Bacillus*, *Massilia*, and *Lysobacter* at root compartments in the T5 stage. Further, transcriptomic analysis revealed that PV-6 enhances drought tolerance in red kidney beans by modulating the expression of genes associated with abiotic stress-related genes. Our findings highlight the potential of *Rhizobium* sp. PV-6 as a bioinoculant for improving drought tolerance in red kidney beans (*Phaseolus vulgaris*), providing a foundation for designing synthetic microbial communities for crop stress resilience.

## 1. Introduction

Red kidney beans (*Phaseolus vulgaris* L.) possess significant nutritional and health-promoting potential, contributing to their increasing cultivation and consumption across various countries worldwide [1,2]. From a nutritional perspective, red kidney beans are notable for their high content of essential amino acids, proteins, complex carbohydrates, vitamins, dietary fibers, minerals, polysaccharides, and peptides, establishing them as a high-quality source of plant protein [3,4]. In terms of health benefits, red kidney beans exhibit properties such as antioxidant, anticancer, antihyperlipidemic, antidiabetic, anti-inflammatory effects, immune modulation, liver protection, neuroprotection, and cardiovascular benefits [4,5]. Notably, the polysaccharides derived from red kidney beans have been the subject of extensive research, particularly with regard to their influence on gut microbiota, which is highly relevant to diabetes management [6,7]. Despite the increasing recognition of the nutritional and health benefits of red kidney beans, it was earlier reported that red kidney beans have limited resistance to abiotic stress, especially under drought conditions [8]. Therefore, enhancing drought resistance and tolerance to other forms of abiotic stress is crucial for the successful cultivation and industrial application of red kidney beans.

Drought stress is considered to be one of the most severe abiotic challenges confronting global crops, with significant potential to threaten agricultural productivity and food security on a worldwide scale [9,10,11]. Projections suggest that by 2050, drought will have a negative impact on 50% of the world’s arable land [12]. In response to such conditions, plants rapidly accumulate reactive oxygen species (ROS) as a primary defense mechanism [13,14,15]. ROS function in a dual capacity in the plant’s response to drought stress; at low levels, they act as signaling molecules that mediate a subset of stress responses, while elevated concentrations of ROS pose a substantial threat to plant survival [16,17]. ROS have been shown to damage macromolecules, including proteins, lipids and nucleic acids, ultimately leading to cell death [18]. Plants have developed a variety of antioxidants that are essential for detoxifying ROS under stress conditions. These include superoxide dismutase (SOD), ascorbate peroxidase (APX), and catalase (CAT), which function by eliminating excess ROS and protecting plants from damage caused by abiotic stresses such as drought, salinity, heat, and cold [19,20,21,22]. Meanwhile, in response to drought stress, plants synthesize abscisic acid (ABA) in various organs, with mesophyll tissues demonstrating a greater capacity for ABA production compared to root tissues [23,24]. The subsequent accumulation of ABA activates signaling pathways that enhance the water use efficiency of crops under drought stress. This process includes the activation of *PYR/PYL/RCAR* (Pyrabactin Resistance 1/PYR1-Like/ABA receptor Regulatory Component of ABA receptor) and *SnRK2* (SNF1-related protein kinase 2) functions, while simultaneously inhibiting the negative regulator *PP2C* (Clade A type 2C protein phosphatase), thereby improving abiotic stress tolerance in plants [25,26,27,28]. Additionally, brassinosteroids have been shown to modulate crop responses to drought stress through ABA-related signaling pathways [29,30,31]. Additionally, BRASSINOSTEROID INSENSITIVE 2 (*BIN2*), a negative regulator of brassinosteroid signaling, is dephosphorylated by ABA INSENSITIVE 1 (*ABI1*) and ABA INSENSITIVE 2 (*ABI2*). ABA activates downstream *BIN2* by inhibiting the activities of both *ABI1* and *ABI2* [32]. The activated *BIN2* subsequently phosphorylates *SnRK2s*, triggering a cascade of downstream pathways [33]. Furthermore, activated *BIN2* has been demonstrated to phosphorylate and stabilize *TINY*, which in turn promotes ABA-induced stomatal closure, thereby enhancing plant drought resistance [34].

In response to drought conditions, plants exhibit alterations in their microbial community structure, enabling them to better cope with the imposed stress [23,35,36,37]. The influence of variations in soil moisture on the associated microbial communities in plants has been shown to be significant [38]. Soil harbors a diverse array of microorganisms, with an average density ranging from 10^8^ to 10^9^ microbial individuals per gram [39]. Interactions among these microbial communities collectively regulate traits associated with crop growth, disease resistance, and stress tolerance [40]. Among these communities, those that promote plant growth are designated as plant growth-promoting rhizobacteria (PGPR) [41]. Previous studies have demonstrated that PGPR enhance plant growth and mitigate drought stress through mechanisms such as hormone production, modulation of root structure, activation of antioxidant defense systems, and induction of stress-responsive gene expression [42,43,44,45]. Consequently, an increasing number of researchers are focusing on the modification of crop microbial communities as a strategy for sustainable food production. This approach offers several advantages over traditional breeding methods, including shorter development cycles, rapid market adoption, and reduced investment costs [46,47,48].

The taxonomic classification of rhizobia as a significant group of bacteria within the category of plant growth-promoting rhizobacteria (PGPR) is well-established. These bacteria form symbiotic relationships with leguminous crops, resulting in the formation of root nodules that convert atmospheric N2 into ammonia, a form that is readily absorbable by plants. This process effectively supplies nitrogen in environments with low nitrogen availability. Furthermore, rhizobia have been shown to play a pivotal role in regulating crop responses to both biotic and abiotic stresses [49,50,51,52]. In a study conducted by Bai et al., the protective effects of 224 bacterial isolates from Arabidopsis leaves were evaluated under sterile conditions. The study found that 18 strains (8%) exhibited strong protective capabilities, four of which belonged to the genus *Rhizobium*. Subsequent investigations utilizing comparative genomics and genetic approaches identified that the protective abilities of various rhizobial strains may be regulated by components of the Type VI secretion system (T6SS) [53]. Moreover, co-inoculation of Rhizobium with arbuscular mycorrhizal fungi under drought stress has been demonstrated to have a positive effect on pod number, seed fresh weight, and seed dry weight in comparison to the control [54]. For variety, inoculation with *R. alamii* GBV030 enhanced the drought tolerance of rapeseed, leading to increased biomass under water stress conditions. Also, the inoculation altered the structure of the root endophytic and rhizosphere microbial communities, which subsequently contribute to increased rapeseed biomass in drought stress environments [55].

This study elucidates the transcriptional and microbial mechanisms underlying the *rhizobium*-induced enhancement of drought tolerance in red kidney beans. The findings provide both theoretical and molecular evidence for the role of *rhizobium* inoculation in improving crop drought stress tolerance, thereby offering new insights for the development of synthetic communities (SynCom).

## 2. Materials and Methods

### 2.1. Plant Materials and Growing Conditions

The red kidney bean variety used in this study was the high-quality cultivar “PinJinYun No. 3” (red kidney bean), bred by Shanxi Agricultural University (Shanxi Academy of Agricultural Sciences) in China. The *Rhizobium* strain selected for the study was the strain designated PV-6, which was isolated from the root nodules of “PinJin Yun No. 3” (Figure S1). Soil samples were collected from the Kelan kidney bean breeding center. The collected soil samples were air-dried at room temperature and then screened using a circular sieve (50 cm diameter, 10 cm height, 5 mm hole spacing). The sieved soil was mixed with sterilized 1–3 mm sand (autoclaved at 121 °C for 15 min) at a 10:1 ratio. The 25 kg resultant soil mixture was placed into pots measuring 60 cm × 30 cm × 18 cm. Fifty-four uniform and plump red kidney bean seeds were sown evenly across each pot. Four treatments were established: W-NO (watering without inoculation of *rhizobium*), W-RHI (watering with inoculation of *rhizobium*), D-NO (drought without inoculation of *rhizobium*), and D-RHI (drought with inoculation of *rhizobium*), with three replicates for each treatment. Based on the previous preliminary experiments. After sowing, each W-RHI and D-RHI pot received 2.5 L of *Rhizobium* PV-6 suspension (OD600 = 0.06–0.08), while W-NO and D-NO pots received 2.5 L of distilled water. No drought treatment was administered between T1 (7 days) and T2 (14 days). For normal watering and rewatering treatments, each pot received 1–2 L of water per application to maintain moist soil without waterlogging. For the drought treatment samples, the bending of the growth point of the top stem of red kidney beans serves as the criterion. Each pot should be watered with an equal amount of 500 mL per application. The plants were cultivated in a greenhouse at 28 °C, with a light cycle of 16:8 h, with a relative humidity of 60–80%, and a photosynthetic photon flux density (PPFD) of 300 µmol m^−2^ s^−1^. Pots were repositioned every two days in a cyclical manner to minimize positional effects. At T1 (after seeding of 7 days), perform thinning to eliminate weak and diseased seedlings.

### 2.2. PV-6 Isolation and Assessment of Strain Nodulation Efficiency

At the Kelan kidney bean breeding center of Shanxi Agricultural University (Shanxi Academy of Agricultural Sciences) in China. Select healthy plants without pests or diseases. Use a shovel to carefully extract the entire root system and gently shake off the soil attached to the roots. Place the roots in a sealed bag on ice for preservation. Upon returning to the laboratory, rinse the roots under running water for 2–3 min to remove visible soil particles. Then, in a laminar flow hood, use sterile tweezers to detach the root nodules and immerse them in a 4% sodium hypochlorite solution for 1 min.

Afterward, transfer the nodules to 75% ethanol and soak them three times for 1 min each to surface-sterilize the nodules. Subsequently, wash the nodules three times with sterile distilled water for 1 min each to remove any residual sodium hypochlorite and ethanol. Finally, use a sterile scalpel and tweezers to bisect the nodules, place the cut surface facing down on a Petri plates with Yeast Extract Mannitol Agar (YEMA) media with Congo red (CR) dye (25 μg mL^−1^), and incubate at 28 °C for 3–5 days [56].The nodulation efficiency of the strains was evaluated by inoculating 14-day-old red kidney bean plants grown in sterilized vermiculite with three individual rhizobial strains (OD600 = 0.08), followed by quantification of root nodules 14 days after inoculation.

### 2.3. Sample Collection

The entire root system was excavated and vigorously shaken to remove loosely attached soil. Extraneous materials were removed, and the soil was thoroughly mixed to homogeneity. A 10 g portion of this sample was then placed in a 15 mL sterile tube, frozen in liquid nitrogen, and subsequently stored at −80 °C. Subsequently, root segments (2–3 cm below the stem-root junction) with visible nodules removed were collected in a 50 mL sterile tube, which was filled with 15 mL of sterile Phosphate-Buffered Saline (PBS). After vortexing for 1 min, the resulting slurry was collected as the rhizosphere sample. To obtain endophytic samples, the vortexed roots were immersed in a 4% sodium hypochlorite solution for 1 min. Subsequently, the roots were transferred to 75% ethanol for a further minute, followed by three 1 min sterile distilled water rinses, to ensure the removal of any residual sodium hypochlorite and ethanol from the red kidney bean roots. Following this, the roots were dried on sterile filter paper to eliminate surface moisture and placed in a sterile 15 mL centrifuge tube. The tubes were flash-frozen in liquid nitrogen and stored at −80 °C for subsequent analysis [57]. To examine the potential growth-promoting and drought tolerance-enhancing effects of PV-6 inoculation on red kidney beans, four experimental treatments were established: W-NO, W-RHI, D-NO, and D-RHI. Sampling was initiated when the second true leaf had fully expanded (T1, after seeding of 7 days). Subsequent samples were collected weekly until T7, covering a total experimental period of 49 days. Between T1 (7 days) and T2 (14 days), no drought stress was imposed to allow normal root development under conditions simulating field environments. Drought treatment was applied to the D-NO and D-RHI groups from T3 (21 days) to T5 (35 days), after which regular watering was resumed at T6 (42 days) and maintained through T7 (49 days) (Figure S2).

### 2.4. Measurement of Enzyme Activity of Superoxide Dismutase (Sod), Peroxidase (POD), Catalase (CAT), and the Content of H_2_O_2_

The activities of the enzymes peroxidase (POD), superoxide dismutase (SOD) and catalase (CAT), as well as hydrogen peroxide (H_2_O_2_) content in leaves short-term (T3) and long-term (T5) drought treatments (D-NO and D-RHI), were measured using the BC0090, BC0170, BC0200, BC3590 kits (Solarbio, Beijing, China) according to the manufacturer’s instructions. The second fully expanded leaf was collected for the enzyme activity and the content of H_2_O_2_ measurements.

### 2.5. DNA Extraction and 16S rRNA Amplicon Sequencing

In total, 252 samples (84 root, 84 rhizosphere and 84 bulk soil samples) were used for sequencing. We treated the frozen root samples stored at −80 °C with bead beating prior to DNA extraction. For this process, we used 0.35–0.4 g of ground up root samples, 1 mL of solution from the rhizosphere (muddy water), and 0.15–0.2 g of soil for DNA extraction. Microbial DNA extraction was performed using the E.Z.N.A.^®^ Soil DNA Kit (Omega Bio-Tek Inc., Norcross, GA, USA). Primers 799F and 1193R were utilized to amplify the highly variable V5–V7 region of the bacterial 16S rRNA gene. The concentration of the extracted DNA was then quantified using the Qubit dsDNA HS Assay Kit and the Qubit 4.0 fluorometer (Invitrogen, Thermo Fisher Scientific, Waltham, MA, USA). Forward primer 779F (AACMGGATTAGATACCCKG) and reverse primer 1193R (ACGTCATCCCCACCTTCC) were utilized to amplify and Illumina PE300 platform sequence the V5–V7 region of the 16S rRNA gene. Both primers were labeled with sample-specific Illumina indexing sequences for subsequent analysis. The subsequent steps included PCR amplification and purification of the amplicons, which were performed in accordance with the methods outlined by Zhang et al. [57]. The constructed library was then subjected to sequencing using the Illumina NovaSeq 6000 platform (Illumina, San Diego, CA, USA). The sobs alpha-diversity was analyzed using the non-singleton amplicon sequence variant (ASV) table in QIIME 2 (2023. 5) and visualized in line charts. β–Diversity (between-sample diversity) was performed using Bray–Curtis distance. Sequence data has been uploaded into the Genome Sequence Archive (GSA) database with the Accession No: CRA022322, https://ngdc.cncb.ac.cn/gsa/search?searchTerm=CRA022322 (accessed on 16 January 2025).

The full-length 16S rRNA genes of three rhizobial strains, PV-6, PV-8, and PV-30, were amplified using the forward primer 27F: AGAGTTTGATCMTGGCTCAG and the reverse primer 1492R: GGTTACCTTGTTACGACTT. The PCR products from these strains were recovered using the DP208 Agarose Gel DNA Recovery Kit (TIANGEN, Beijing, China). Subsequently, the PCR products were sequenced using the VT402 flat-end plus A cloning kit (TIANGEN, Beijing, China). The 16S rRNA gene sequences of PV-6, PV-8, and PV-30 are provided in Table S3.

### 2.6. qRT-PCR Gene Expression and Transcriptome Analysis

The RNA extraction process from red kidney bean roots was performed using the RNAprep Pure Plant Plus Kit (TIANGEN, Beijing, China) at T5 stage, across W-NO, W-RHI, D-NO, D-RHI treatments [44], three biological replicates were performed for each treatment. The concentration of the extracted total RNA was then quantified using the Qubit dsDNA HS Assay Kit and the Qubit 4.0 fluorometer (Invitrogen, Thermo Fisher Scientific, Waltham, MA, USA). Samples from the T5 stage that met the criteria for transcriptome analysis were subsequently analyzed by Meiji Biological Company (Majorbio, Shanghai, China). Following the completion of library construction, RNA sequencing was applied on the Illumina NovaSeq 6000 platform (Illumina, San Diego, CA, USA). The differential gene expression analysis was conducted on the sample groups using DESeq2 software, with the criteria for differential gene selection established at |log2(Fold Change)| ≥ 1 and padj ≤ 0.05. Gene Ontology (GO) enrichment analysis was then performed for the differentially expressed genes, considering terms with padj < 0.05 as significantly enriched. Additionally, Kyoto Encyclopedia of Genes and Genomes (KEGG) enrichment analysis was carried out for differentially expressed genes (padj < 0.05) to identify the metabolic and signaling pathways associated with these genes.

The relative expression levels of five drought stress-related marker genes at the T5 stage were evaluated using quantitative real-time PCR (qRT-PCR). The RNA was reverse transcribed into cDNA utilizing the HiScript II Reverse Transcriptase Kit (Vazyme Biotech, Nanjing, China). The qRT-PCR was then conducted on a 96-well plate employing the SYBR Green PCR Kit (Toyobo, Osaka, Japan). *PvActin* served as the internal reference, and relative expression levels were analyzed using the 2^−ΔΔCt^ method. The qRT-PCR primers are provided in Table S2.

### 2.7. Statistical Analyses

Graphical representations were generated with GraphPad Prism 5 (GraphPad Software, Inc., La Jolla, CA, USA). All results obtained are presented as the mean ± standard deviation from three independent experiments. A phylogenetic tree was constructed using MEGA 5 with the neighbor-joining method with the bootstrap value at 1000 repetitions. The visualization of phylogenetic tree is performed through ITOL (V7. 2. 1) [58]. Asterisks indicate statistical significance in one-way or two-way analysis of variance (* *p* < 0.05; ** *p* < 0.01; *** *p* < 0.001), while “N.s.” represents no significant difference. Each experiment was performed with three biological replicates. Taxonomic assignment of Amplicon Sequence Variants (ASVs) was conducted using the Naive Bayes consensus taxonomy classifier implemented in QIIME 2 in conjunction with the SILVA 16S rRNA database (version 138). Various analyses and functional predictions, including Spearman’s correlations, species composition analysis, and species difference analysis, were performed on the Majorbio Cloud Platform https://www.majorbio.com/tools (accessed on 21 July 2023), a free online resource.

## 3. Results

### 3.1. PV-6 Enhances Plant Growth and the Activity of Antioxidant Enzymes of Red Kidney Beans

We isolated three *rhizobium* strains from the root nodules of Pinjinyun No. 3 red kidney bean plants, designated as PV-6, PV-8, and PV-30. 16S rRNA sequence BLAST (2. 17. 0) analysis and phylogenetic tree construction revealed that PV-6 and PV-30 probably belong to the *Rhizobium leguminosarum* species complex [59]. Phylogenetic analysis demonstrated a close relationship between PV-6 and PV-30 (Figure S1E). Subsequently, we assessed the nodulation efficiency of the three *rhizobium* strains. After seeding of 7 days inoculation with three *rhizobium* strains (OD600 = 0.08), 14 days post-inoculation, strain PV-6 exhibited a significantly higher number of nodules compared to both PV-8 and PV-30 (Figure S1A–D), Consequently, PV-6 was selected for use in subsequent experiments.

**Figure 1:**
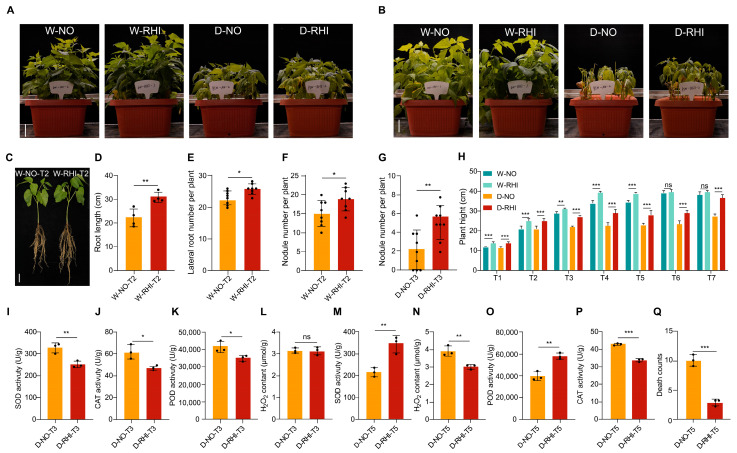
Drought tolerance of red kidney beans was enhanced through the inoculation process with PV-6. (**A**): Growth phenotype of red kidney beans at T3 stages under four treatments: W-NO, W-RHI, D-NO, and D-RHI. The scale bar = 5 cm. (**B**): Growth phenotype of red kidney beans at T5 stages under four treatments: W-NO, W-RHI, D-NO, and D-RHI. The scale bar = 5 cm. (**C**): Representative image showing the root phenotype of red kidney beans 14 days after inoculation with PV-6 compared to the uninoculated control. Scale bar = 5 cm. (**D**–**F**): Root length, number of lateral roots, and nodule count at 14 days with inoculation PV-6. Three plants were randomly selected per treatment, with each experiment repeated three times. Asterisks indicate statistically significant differences between PV-6 inoculated plants and controls. (**G**): Nodule count under short-term drought stress at T3 stage in D-NO and D-RHI treatments. Statistical significance is denoted by asterisks. (**H**): Plant height at seven time points under four treatments. Asterisks indicate significant differences between PV-6 inoculated plants and controls. (**I**–**P**): Enzyme activities of SOD, CAT, POD, and H_2_O_2_ content in leaves under short-term drought stress at T3 stage and long-term drought stress at T5 stage in D-NO and D-RHI treatments. Significant differences are indicated as. SOD, superoxide dismutase; CAT, catalase; POD, peroxidase; H_2_O_2_, hydrogen peroxide. (**Q**): Surviving rate After long-term drought stress at T5 stage. Statistical significance between treatments is indicated by asterisks. * *p* < 0.05, ** *p* < 0.01, *** *p* < 0.001, ‘ns’ indicates no significant difference.

Next, root length, lateral root count and root nodule quantity of red kidney beans in different treatment groups at the T2 stage we conducted an analysis of the results show that inoculation with PV-6 significantly increased root length, lateral root number and nodule number in red kidney beans at stage T2 compared to the control (Figure 1C–F). Statistical analysis of aboveground traits across seven time points and four different treatments indicated that inoculated plants exhibited significantly greater plant height, fresh weight, and dry weight than the uninoculated control Figure 1H and Figure S3C,D).

As well established, rhizobia enhance legume growth through atmospheric nitrogen fixation [60]. Therefore, we hypothesized that inoculating PV-6 would enhance nitrogen fixation and improve the drought stress tolerance of red kidney beans. Seven days after sowing the red kidney beans, drought stress was induced by withholding water, using 100 mL of distilled water as the control. Our findings indicate that the treatment with 100 mL of 15.75 mmol/L KNO_3_ alleviated wilting in red kidney beans and improved their tolerance to drought stress (Figure S1F).

We subsequently measured the soil moisture content at each sampling period. This is related to the available water volume of plant roots [38]. Soil moisture content analysis revealed that the D-NO and D-RHI treatments significantly reduced soil moisture compared with the W-NO and W-RHI treatments at stages T3, T4 and T5. Moreover, soil moisture levels remained consistent between D-NO, D-RHI treatments during the same intervals (Figure S3A). Notably, inoculated PV-6 plants demonstrated superior growth performance relative to their uninoculated PV-6 counterparts. Under drought conditions, the D-RHI plants exhibited reduced leaf curling and wilting in comparison to the D-NO treatment (Figure 1A,B). Finally, after the treatment of drought stress at T5, we found that the survival rates of red kidney beans subjected to D-RHI treatments increased by 20.5% compared to the D-NO control (Figure 1Q). These results conclusively demonstrate that PV-6 inoculation enhances the drought tolerance of red kidney beans.

Plants have evolved diverse strategies to cope with drought stress, including activating oxygen scavenging mechanisms to mitigate cellular toxicity from reactive oxygen species (ROS) accumulation during drought. In order to assess the activity of antioxidant-related enzymes in red kidney beans subjected to drought stress with or without PV-6 inoculation, the activities of catalase (CAT), superoxide dismutase (SOD), and peroxidase (POD), along with hydrogen peroxide (H_2_O_2_) content and nodule number at T3 and T5 stages. As demonstrated in Figure 1 at the T3 stage, the activities of leaf CAT, SOD, and POD in the drought-inoculated treatment were significantly reduced compared to the control. Notably, no significant differences were observed in H_2_O_2_ contents. The nodule number in the D-RHI treatment was significantly higher than those in the uninoculated control (Figure 1G,I–L). At the T5 stage, the activities of SOD and POD in the leaves of the D-RHI treatment were significantly increased compared to the D-NO control, while CAT activity and H_2_O_2_ content were significantly decreased (Figure 1M–P). In summary, the findings of this study demonstrate that PV-6 inoculation enhances the drought stress tolerance of red kidney beans by modulating the activity of antioxidant enzymes.

### 3.2. Dynamic Shifts in the Dominant Taxa of Red Kidney Beans When Subjected to Different Treatments

Microbial communities and beneficial microorganisms have been shown to play a crucial role in enhancing plant drought tolerance [61,62]. In order to investigate the microbial composition and diversity in red kidney beans under drought conditions, with and without the inoculation of PV-6, bulk soil, rhizosphere, and root samples were collected from the W-RHI, W-NO, D-RHI, and D-NO treatments across all seven stages. The V5–V7 regions of the 16S rRNA were amplified and sequenced. Among the total of 252 samples, 19,153,921 high-quality sequences were identified, yielding an average of 76,008 sequences per individual sample (ranging from 43,050 to 228,173 bp), which amounts to a cumulative total of 7,219,799,730 bp and the average read length of the inferred amplicon sequence variants (ASVs) of 377 bp (ranging from 200 to 532 bp). Following the rarefaction of the sequencing data, the total number of Amplicon Sequence Variants (ASVs) was reduced to 634,884, with an average sequence count of 24,729 per individual sample, leading to the identification of 504,857 bacterial ASVs (Table S1). To assess the reliability of the data, an alpha diversity rarefaction curve analysis was conducted. The results demonstrated that as the sequencing depth increased, the number of detectable new bacterial ASVs gradually decreased, suggesting that our sequencing data encompassed the majority of the bacterial ASVs present in the analyzed samples (Figure S3B).

To elucidate the effects of drought stress, inoculation of PV-6, and their combined effects, as well as the temporal dynamics on the structure of bulk soil, rhizosphere, and root bacterial communities in red kidney beans, the abundance of bacterial communities was analyzed at both the phylum and genus levels across seven stages and four treatments. Relative abundance analysis indicated that the soil bacterial communities demonstrated a high degree of stability across all samples (Figure S4A,B), which is consistent with previous studies [38,63]. At the phylum level, the bacterial communities of the rhizosphere exhibited comparable dynamic changes over time across four treatments (Figure S4C). However, at the genus level, The W-RHI treatment experienced a more Intense alteration in the abundance of various bacterial genera compared to the other three treatments (Figure S4D). This finding suggests elevated competitive interactions among bacterial communities within the W-RHI group.

**Figure 2:**
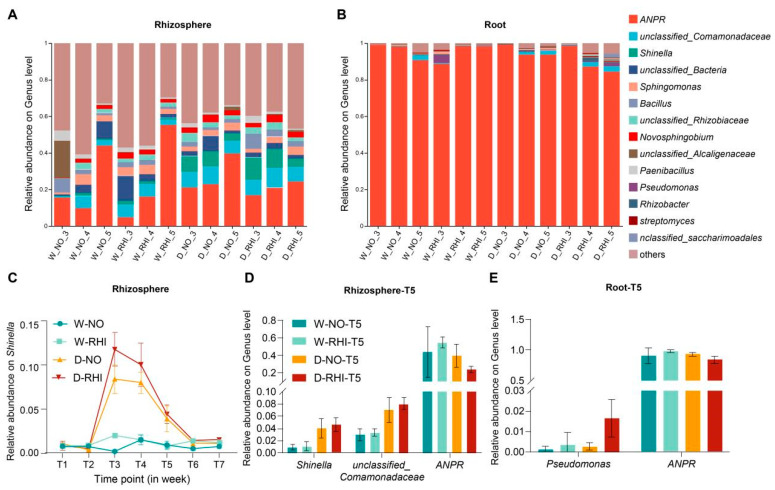
Changes in microbial community abundance in red kidney beans under different treatments. (**A**,**B**): The relative abundance of the top 10 most abundant Genus in the rhizosphere and root at T3–T5 stages under four treatments: W-NO, W-RHI, D-NO, and D-RHI. Unresolved taxa as “unclassified” was labeled. The top ten bacterial genera with the highest abundance are displayed. The category labeled ‘others’ includes bacterial genera that are not among the top ten. (**C**): Changes in the relative abundance of Shinella in the rhizosphere at seven time points under four treatments: W-NO, W-RHI, D-NO, and D-RHI. (**D**,**E**): Microbial abundance at the Genus level in the rhizosphere and root at T5 stage under four treatments: W-NO, W-RHI, D-NO, and D-RHI.

Distinct differences in the abundance of various bacterial genera were observed between the watering and drought treatments at the T3 to T5 stages, particularly regarding differential abundance of *Shinella*. In contrast, at the T1 and T2 stages, *Shinella* exhibited low abundance levels in the rhizosphere across all four treatments. However, during the T3–T5 periods of drought stress, the abundance of *Shinella* in the rhizosphere of red kidney beans increased rapidly. Following rehydration at the T6 stage, the abundance of *Shinella* returned to levels comparable to those observed in the watering treatments (Figure 2A and C). Consequently, we hypothesize that *Shinella* may serve as a marker bacterium for plants’ response to earlier drought stress. At the subsequent drought stress stage (T5), the relative abundances of *Allorhizobium-Neorhizobium-Pararhizobium-Rhizobium*, *Shinella*, and *unclassified_Comamonadaceae* exhibited distinct abundance differences between the drought and watering treatments, suggesting that the bacterial communities at the genus level in the rhizosphere of red kidney beans are greater sensitivity to drought stress compared to those subjected to inoculation (Figure 2D). *Allorhizobium-Neorhizobium-Pararhizobium-Rhizobium* is a group composed of four different rhizobia genera. For easier expression, we abbreviate it as *ANPR* [64]. Additionally, the abundance of root endophytic bacterial communities at both the phylum and genus level in the W-NO, D-NO, and D-RHI treatments demonstrated a similar temporally dynamic distribution. However, the W-RHI treatment exhibited consistently higher abundance of Proteobacteria across all seven stages (Figure S4E,F). Meanwhile, in the D-RHI treatments of the three periods of T3–T5, the abundance of ANPR showed a gradually decreasing trend compared with the other three treatments, reaching its lowest abundance level in T5, while the abundance of Pseudomonas increased (Figure 2B,E).

### 3.3. Drought Stress Alters the Microbial Community Diversity of Red Kidney Beans

**Figure 3:**
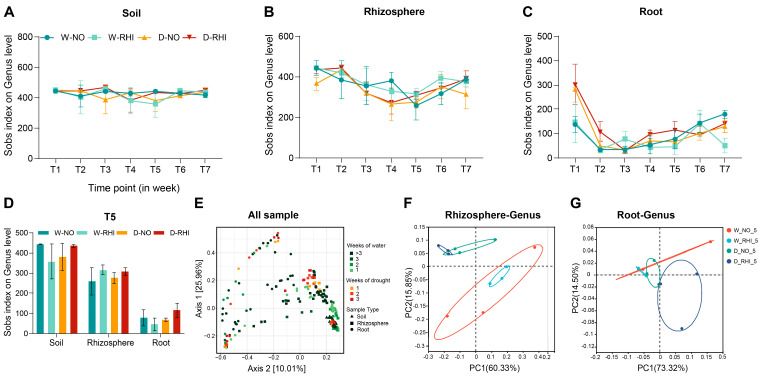
Effects of D-NO and D-RHI on microbial diversity in the rhizosphere and root. (**A**–**C**): Changes in microbial α-diversity in soil, rhizosphere, and root across seven time points under four treatments: W-NO, W-RHI, D-NO, and D-RHI. (**D**): Differences in microbial α-diversity at T5 stage in soil, rhizosphere, and root among the four treatments: W-NO, W-RHI, D-NO, and D-RHI. (**E**): Principal Coordinates Analysis (PCoA) of Bray–Curtis distances for microbial communities in soil, rhizosphere, and root samples at seven time points under the four treatments: W-NO, W-RHI, D-NO, and D-RHI. (**F**,**G**): PCoA of Bray–Curtis distances illustrating microbial community differences in the rhizosphere and root at T5 stage across the four treatments: W-NO, W-RHI, D-NO, and D-RHI.

It has previously been demonstrated that an increase in the diversity of the bacterial community under conditions of drought stress may result in an elevated frequency of functional genes within these bacterial communities, thereby enhancing the drought resistance of crops [65,66]. To investigate this hypothesis, an analysis of the α-diversity (Sobs index) at the genus level was conducted for seven stages across four treatments and three ecological niches. However, at three stages of drought stress (T3–T5), no significant changes were observed in the rhizosphere and soil bacterial communities’ diversity across the four treatments (Figure 3A,B). Even at the T5 stage, the community diversity among the four treatments remained stable (Figure 3D). In contrast, the root bacterial communities, predominantly composed of Proteobacteria and *ANPR*, exhibited substantial differences in bacterial community diversity. It is noteworthy that, The microbial community diversity under T5 D-RHI treatment was significantly higher than W-NO, W-RHI, D-NO (Figure 3C,D). These results suggested that the bacterial community diversity varied among the red kidney bean T5 stage roots in the four treatments showed greater variation than in the rhizosphere or bulk soil samples (Figure 3D).

PCoA at the genus level indicated that the bacterial communities in bulk soil maintained a high level of stability across all treatments, while the rhizosphere bacterial communities were significantly affected by drought stress, exhibiting distinct differences in community distribution between drought and watering samples. The most significant variations in distribution were exhibited by the root bacterial communities in response to drought stress (Figure 3E). To elucidate the differences in rhizosphere and root endophytic bacterial communities under drought stress, PV-6 inoculation, and their combined during T5 period, we performed a *β*-diversity PCoA at the genus level across the four treatments. The rhizosphere bacterial communities exhibited positional differences between different treatments (differences in distribution). However, these differences seem to be less affected by PV-6 inoculation (Figure 3F). PCoA of the root bacterial communities revealed that neither PV-6 inoculation nor drought stress affected their spatial distribution. However, a notable difference in positional distribution was observed under the combined treatment of drought and inoculation (Figure 3G). These results suggest that the rhizosphere is more sensitive to drought stress than PV-6 inoculation. Conversely, root endophytic bacterial communities showed no response to either treatment individually, displaying a distinct community structure only under the interaction of drought stress and PV-6 inoculation.

### 3.4. PV-6 Inoculation Reshaped the Root Bacterial Community

**Figure 4:**
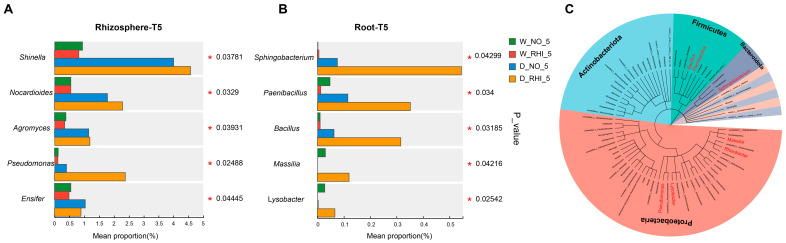
Reshaping of the root microbiome in red kidney beans due to drought with inoculation treatment. (**A**,**B**): The top five bacterial taxa exhibited significant differences among the four treatments (W-NO, W-RHI, D-NO, D-RHI) at T5 stage in both the rhizosphere and root. Asterisks denote significant differences (* p < 0.05). (**C**): Phylogenetic tree of microbial communities at the genus level in soil, rhizosphere, and root across seven time points. The internal colors represent phyla, with red indicating microbes that were enriched in the D-RHI treatment at T5 stage.

In order to further elucidate the bacterial abundances influenced by PV-6 inoculation, the drought tolerance of red kidney beans, the following experiment was conducted. The top five genera of bacteria that were significantly enriched across four treatments during the T5 stages in rhizosphere and root compartment were selected for further analysis. The results indicate that during the T5 period, significant differences were observed in the enrichment of *Shinella*, *Nocardioides*, *Agromyces*, and *Ensifer* within the rhizosphere, which were influenced by drought stress. Notably, *Pseudomonas* exhibit specific enrichment under D-RHI treatment (Figure 4A). Meanwhile, a specific enrichment of microorganisms such as *Sphingobacterium*, *Paenibacillus*, *Bacillus*, *Massilia*, and *Lysobacter* was identified in the D-RHI roots at the T5 stage (Figure 4B). Furthermore, the microorganisms that specifically enriched in the roots under D-RHI treatments were different from those enriched in the rhizosphere under D-NO and D-RHI. These findings demonstrate that D-NO and D-RHI treatments recruit different microorganisms in the rhizosphere and root of red kidney beans, respectively, and D-RHI treatments specifically reshape the root microbial communities, which may be an important cause for the phenotypic difference between D-NO and D-RHI under drought stress T5 stages. To further investigate these findings, a phylogenetic tree was constructed, encompassing bacterial communities with an abundance (>0.01) in soil, rhizosphere, and root at the genus level. This analysis enabled the examination of the phylogenetic relationships of the D-RHI-specific enriched bacteria (Figure 4C). In summary, PV-6 inoculation under drought stress conditions enabled red kidney beans to recruit specific bacterial communities to address the survival challenges posed by drought.

### 3.5. Drought-Inoculation Treatment Altered the Expression of Drought-Related Genes in Red Kidney Beans

**Figure 5:**
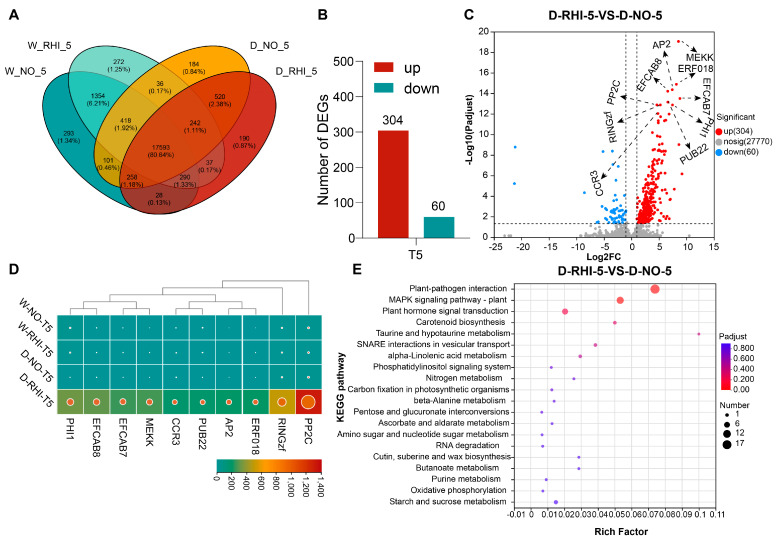
Differential expression of drought-related genes under drought conditions with inoculation treatment. (**A**): Differentially expressed unique and overlapping genes at T5 stage across the four treatments: W-NO, W-RHI, D-NO, and D-RHI. (**B**): The number of upregulated and downregulated genes in the D-NO and D-RHI treatments at T5 stage. (**C**): A volcano plot illustrating the top 10 differentially expressed genes in the D-NO and D-RHI treatments at T5 stage. (**D**): A heatmap displaying the expression levels of the top 10 differentially expressed genes at T5 stage under the four treatments: W-NO, W-RHI, D-NO, and D-RHI. The sizes of the dots in the figure represent the expression levels of the genes. A larger indicates a higher level of gene expression. (**E**): KEGG pathway enrichment analysis of differentially expressed genes in the D-NO and D-RHI treatments at T5 stage.

In order to further elucidate the molecular mechanisms by which the combined treatment of drought and PV-6 inoculation recruits specific bacteria to enhance the drought tolerance of red kidney beans, was conducted a transcriptomic analysis of the roots of red kidney beans subjected to drought stress at the T5 stage was conducted. To assess the usability of the transcriptomic data and determine whether red kidney beans respond to the drought stress environment we provided, we assessed the expression levels of drought stress-related marker genes using qRT-PCR at the T5 stage. The results indicated that the relative expression of *LEA3*, *NCED4* significantly increased under drought stress, whereas the relative expression of *PIP1*, *PIP2* and *P5CS* significantly decreased (Figure S5A,B). This finding is consistent with the transcriptome data, further confirming its reliability [67,68,69,70,71]. At the T5 stage, we employed a Venn diagram to analyze the specific and overlapping gene expression across the four treatments during the T5 period. To assess the differences in gene expression between drought stress and PV-6 inoculation under drought conditions,17,593 genes in common to all conditions were not differentially expressed. We examined the DEGs between D-NO and D-RHI. A total of 364 DEGs were identified, comprising 304 upregulated and 60 downregulated genes (Figure 5A,B).

The KEGG pathway enrichment analysis of the differentially expressed genes (DEGs) revealed significant alterations in various metabolic pathways. At the T5 stage, a significant enrichment of differential metabolic pathways was observed, particularly in plant immunity-related pathways, carbon-nitrogen nutrient metabolism, cutin and wax biosynthesis, RNA degradation, and vesicle transport, all of which are relevant to plant responses to abiotic stress (Figure 5E).

We used the volcano map to label the top 10 genes with the most significant differences in expression, including protein kinases (*MEKK*), PHOSPHATASE (*PP2C*), ethylene response factors (*ERF018*, *AP2*), EF-hand calcium-binding domain proteins, E3 ubiquitin ligases, zinc finger proteins, and phosphohexose isomerase (*PHI*) encoding genes, which have been reported in the regulation of drought stress in other crops (Figure 5C) [72,73,74,75]. Subsequently, a heatmap analysis was conducted to assess the expression of the potential drought stress-related genes in red kidney beans. The results indicated that all DEGs at the T5 stages exhibited significant differences, with a predominant enrichment observed in the D-RHI treatment (Figure 5D). Our transcriptome study showed that PV-6 inoculation increases red kidney beans drought tolerance by modulating the expression of abiotic stress-related genes.

### 3.6. Differentially Enriched Specific Bacteria and the Differentially Expressed Gene Identified by Integrated Association Analysis

**Figure 6:**
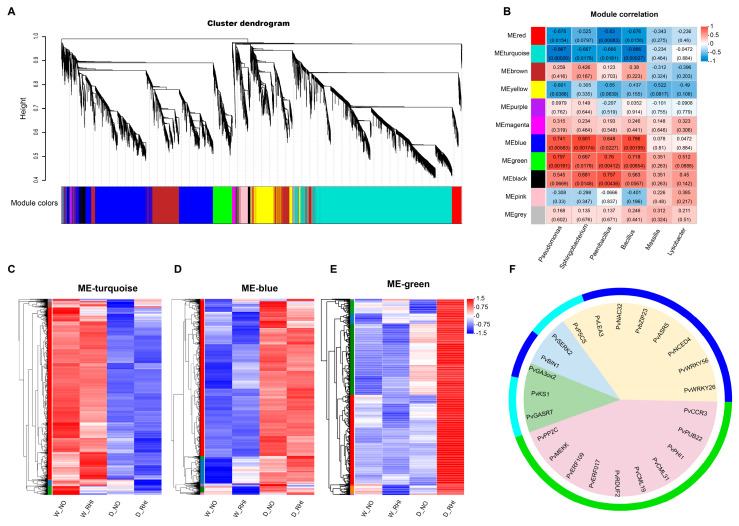
Correlation analysis between specific enriched microbes and the DEGs at T5 stage. (**A**): Clustering diagram of differential expression genes and specific enrichment bacteria in T5 period by WGCNA. (**B**): Correlation analysis of differentially expressed gene modules and differentially enriched root bacteria across the four treatments: W-NO, W-RHI, D-NO, and D-RHI. The data presented in the table represent the correlation coefficients and the significant *p*-value between the modules and microbial genera. (**C**–**E**): Gene expression heatmap for the turquoise, blue, and green modules. (**F**): Analysis of the metabolic pathways to which the differentially expressed drought stress-related genes in the turquoise blue green three modules belong. The internal colors correspond to the signaling pathways: light pink for the top 10 differentially expressed genes at T5 stage under D-NO and D-RHI treatments, light yellow for genes related to the ABA signaling pathway, light green for genes related to the GA signaling pathway, and light blue for genes related to the BR signaling pathway. The outer color bands represent the turquoise blue and green modules to which the related genes belong.

In order to ascertain the correlation between the specific enrichment of *Pseudomonas*, *Sphingobacterium*, *Paenibacillus*, *Bacillus*, *Massilia*, and *Lysobacter* at the T5 stage in the D-RHI treatment and abiotic stress-related genes, a Weighted Gene Co-expression Network Analysis (WGCNA) was conducted on the differentially enriched bacteria and transcriptomic data. The analysis identified 11 distinct modules, with turquoise, blue, and green modules demonstrating significant correlations with the abundances of *Pseudomonas*, *Sphingobacterium*, *Paenibacillus*, and *Bacillus* (Figure 6A,B). The turquoise module comprised 5352 genes, the blue module included 3699 genes, and the green module contained 703 genes. Subsequently, we performed heatmap analyses of gene expression for the genes within these three modules. The results indicated that the majority of the 3699 genes in the blue module were highly expressed under drought stress conditions. Conversely, the 703 genes in the green module were predominantly expressed at high levels in D-RHI treatments. Also, the 5352 genes in the turquoise module exhibited a high expression pattern under watering conditions (Figure 6C–E).

Subsequently, we conducted a KEGG enrichment analysis of genes within these three modules that exhibited the anticipated expression patterns. The results obtained revealed that these gene sets were enriched in pathways related to carbohydrate and starch metabolism, flavonoid metabolism, zeatin biosynthesis, and the plant MAPK signaling pathway, all of which are associated with plant responses to drought stress (Figure S6A–C). Subsequently, the top 10 differentially enriched genes under drought treatment at the T5 stage, along with genes related to ABA, BR, and GA pathways associated with drought tolerance were analyzed that enrichment module analysis. The results indicated that all top 10 genes associated with drought stress were enriched in the green module, while all ABA pathway-related genes, except for P5CS, were enriched in the blue module. Moreover, genes related to the BR and GA pathways were found to be enriched in both the green and turquoise modules (Figure 6F). This finding suggests a predominantly positive role for *Pseudomonas*, *Sphingobacterium*, *Paenibacillus*, and *Bacillus* in enhancing drought tolerance in red kidney beans.

## 4. Discussion

### 4.1. PV-6 Inoculation Positively Regulate Red Kidney Beans Growth

Endophytic bacteria represent a subgroup of plant growth-promoting rhizobacteria (PGPR). These soil bacteria can coexist with plants and enhance crop growth [76,77]. PGPR contribute to increased nutrient acquisition [78,79], improved tolerance to abiotic stress [80], and the induction of crop immunity [81]. However, the mechanisms by which rhizobium inoculation dynamically regulates the growth and development of red kidney beans, particularly in response to drought stress, remain unclear. In this study, we analyzed the phenotypes of red kidney beans across seven stages and four different treatments. Our findings indicate that PV-6 inoculation positively influences the biomass of red kidney beans, including plant height, fresh weight, and dry weight, under both watering and drought stress conditions. Furthermore, after rehydration, the PV-6-inoculated plants exhibited a faster growth rate, with increases in plant height, fresh weight, and dry weight of 96.4%, 105.3%, and 80.9%, respectively, compared to the control group (D-NO).

These findings are consistent with that inoculating rice with *Azorhizobium caulinodans* ORS 571, *Sinorhizobium meliloti* 1021, and *Mesorhizobium huakui* 93 significantly increased root volume, aboveground dry weight, and plant height [82]. Inoculation with drought-resistant rhizobia SRL5 and SRC8, which were isolated from various species, led to increases of 166% in the number of tillers per wheat, 32% in straw yield, and 48% in grain yield under drought stress [83].

### 4.2. PV-6 Inoculation Reshapes the Root Endophytic Bacterial Community in Red Kidney Beans Under Drought Stress

When plants encounter adverse environmental conditions, they often recruit specific bacterial communities to help mitigate these challenges [38,84]. Our KNO_3_ treatment experiment also demonstrated that a single nitrogen treatment can help enhance the drought stress tolerance of red kidney beans. This aligns with the observation that inoculation with PV-6 retains more root nodules under short-term drought stress, thereby potentially improving the drought stress tolerance of red kidney beans by facilitating nitrogen fixation from the air. However, under prolonged drought stress, when all nodules died, our findings indicate that PV-6 inoculation significantly influences the root endophytic bacterial community of red kidney beans under drought stress, leading to increased community diversity. Notably, during the T3–T5 stages, the root endophytic bacterial communities in red kidney beans exhibited a markedly high abundance of *ANPR* across all four treatments. Although the root endophytic bacterial communities exhibited lower diversity compared to soil and rhizosphere bacteria, a significant increase in bacterial community diversity was observed at the T5 stage under drought stress following PV-6 inoculation. This finding suggests that PV-6 inoculation reshapes the root endophytic bacterial community of red kidney beans under drought stress conditions, facilitating the recruitment of specific bacteria to address the challenges posed by drought stress [85,86].

Under prolonged drought stress, the roots of red kidney beans specifically enriched endophytic bacteria such as *Sphingobacterium*, *Paenibacillus*, *Bacillus*, *Massilia*, and *Lysobacter*. These bacteria have been previously reported to play crucial roles in crop responses to drought stress. For instance, *Sphingobacterium* strains isolated from the root system of aloe, when re-inoculated into drought-stressed maize, significantly increased root length, total chlorophyll, total carbohydrates, proline, total protein, total phenolic compounds, and total flavonoid content [87]. The artificial bacterial community SPMX, which consists of *Paenibacillus* amylolyticus and three additional bacterial species, significantly enhances the survival rate of Arabidopsis under drought stress after 21 days. This enhancement is achieved by stabilizing the diversity and structure of root-associated bacterial communities, as well as regulating chlorophyll content and endogenous ABA signaling [88]. Also, *Bacillus pumilus* G5 markedly increases the levels of soluble sugars, soluble proteins, free amino acids, and glycyrrhizic acid by influencing the carbon and nitrogen metabolic processes in *G. uralensis* seedlings. This influence helps to alleviate the adverse effects of drought stress on their growth and development [89]. These observations may be related to the enhancement of drought stress tolerance in red kidney beans through rhizobia re-inoculation [62].

Furthermore, these bacteria influence the activity of crop antioxidant enzymes, thereby regulating drought stress tolerance [90,91,92,93]. This finding further corroborates our results, which demonstrate the enzyme activities of superoxide dismutase (SOD), catalase (CAT), peroxidase (POD), and hydrogen peroxide (H_2_O_2_) content in red kidney beans under drought stress at the T5 stage. Inoculation with the rhizobial strain PV-6 significantly enhanced drought tolerance and increased surviving rate of red kidney beans.

### 4.3. PV-6 Inoculation Altered the Expression of Drought-Related Genes in Red Kidney Beans Under Drought Stress

The recruitment of beneficial microorganisms by crops to regulate gene expression in response to adverse environmental conditions has been extensively studied. In wheat, the primary pathogen responsible for wheat stem rot is *Fusarium graminearum*. However, the inoculation of the root-colonizing bacterium *Sphingomonas* sp. SR80 can mitigate the effects of Fusarium graminearum on wheat by modulating the expression of genes such as *TaAOS*, *TaNPR1*, *Lipase*, *WRKY78*, *PR2*, and *PR3*. This modulation enhances the biomass of both the aboveground and underground parts of the wheat plant, thereby improving seedling survival rates under *Fusarium* inoculation [94]. In soybean, the screening of 1893 microbial strains resulted in the establishment of three synthetic communities (SynComs), which significantly enhanced plant height, biomass, nitrogen content, and phosphorus content compared to control groups, both in field and laboratory settings. Transcriptome sequencing of SynCom1, which showed the most pronounced growth-promoting effects, revealed differential expression of numerous genes associated with nitrogen and phosphorus uptake and transport, symbiotic nodule formation, auxin signaling, and photosynthesis [95].

In our study, PV-6 inoculation exhibited differential gene expression under both drought and non-drought stress conditions. Notably, at 14 days after inoculation (DAI), the regulated changes in root length and the number of lateral roots in red kidney beans. The top ten DEGs at the T5 stage were exclusively related to drought. This observation suggests that the drought treatment, in conjunction with the inoculation of rhizobia, induced a more pronounced expression of plant stress-related genes compared to the control. Consequently, this enhanced expression may be formed by the red kidney beans to recruit beneficial microorganisms, thereby mitigating adverse effects of drought stress.

## 5. Conclusions

**Figure 7:**
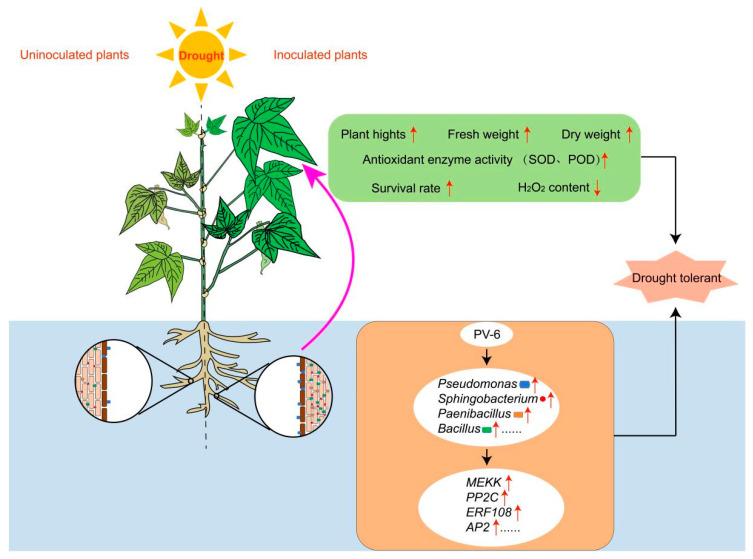
Nodulation reshaped the root microbiome in red kidney beans under drought conditions. The PV-6 treatment modified the root microbiome by selectively recruiting *Pseudomonas*, *Sphingobacterium*, *Paenibacillus*, *Bacillus*, and other microbial species under prolonged drought stress. This modification has been shown to regulate the differential expression of drought-related genes, increase leaf superoxide dismutase (SOD) and peroxidase (POD) enzyme activities, as well as plant height, fresh weight, dry weight, and survival rate, while simultaneously reducing leaf hydrogen peroxide (H_2_O_2_) content. Collectively, these effects have been demonstrated to enhance drought tolerance.The red arrows in the figure indicate the increase or decrease of the represented physiological indicators, microbial abundance, and gene expression levels.

In summary, the present study demonstrates that PV-6 inoculation enhances the biomass of both the aboveground and underground parts of red kidney beans, as well as their tolerance to drought stress. During the early growth stage under well-watered conditions, PV-6 inoculation alters root structure, establishing a solid foundation for drought tolerance in red kidney beans. However, under prolonged drought T5 stage, PV-6 inoculation has been observed to modify the root endophytic microbial community in red kidney beans in comparison to W-NO, W-RHI, and D-NO. This outcome can be attributed to the potential drought-resistant microorganisms from the rhizosphere and roots, including *Pseudomonas*, *Sphingobacterium*, *Paenibacillus*, and *Bacillus*. These microorganisms have been observed to influence the expression of drought-related genes and the activity of antioxidant enzymes, such as superoxide dismutase (SOD), catalase (CAT), and peroxidase (POD), while also reducing hydrogen peroxide (H_2_O_2_) levels. Consequently, they enhance the tolerance of red kidney bean plants exposed to prolonged drought stress and increase their survival rates (Figure 7).

## Data Availability

The data presented in this study are openly available in Genome Sequence Archive at https://ngdc.cncb.ac.cn/gsa/search?searchTerm=CRA022322 (CRA022322) (accessed on 16 January 2025).

## Supplementary Materials

The following supporting information can be downloaded at: https://www.mdpi.com/article/10.3390/microorganisms13092153/s1, Table S1: Amplicon sequence variants (ASVs) of 252 samples; Table S2: Genes colone and qRT-PCR Primer sequences; Table S3: 16s rRNA gene sequence; Figure S1: PV-6 strain had the best combining ability with “PinJinYun No. 3”; Figure S2: Red kidney bean experimental treatment diagram; Figure S3: Nodulation enhances plant growth and drought stress tolerance of Red kidney beans; Figure S4: Microbial community of red kidney bean were changed in Phylum and Genus level; Figure S5: Drought relation marker genes expression in T5 period; Figure S6: Modules KEGG pathway enrichment.
