# Supplementary material for: Rhizobium-Enhanced Drought Tolerance in Red Kidney Beans Through Modification of Transcriptome and Microbial Communities
Source: Microorganisms. 2025 Sep 16;13(9):2153. doi: 10.3390/microorganisms13092153 (PMC12472484; doi:10.3390/microorganisms13092153)
Supplement: Supplementary file 1 [file microorganisms-13-02153-s001.zip › microorganisms-3838642-supplementary.pdf]

Supplementary data

Figure S1. PV-6 strain had the best combining ability with "PinJinYun No. 3"
Figure S2. Red kidney bean experimental treatment diagram
Figure S3. Nodulation enhances plant growth and drought stress tolerance of Red kidney beans
Figure S4. Microbial community of red kidney bean were changed in Phylum and Genus level
Figure S5. Drought relation marker genes expression in T5 period
Figure S6. Modules KEGG pathway enrichment
Table S1. Amplicon sequence variants (ASVs) of 252 samples
Table S2. Genes colone and qRT-PCR Primer sequences
Table S3. 16s rRNA gene sequence

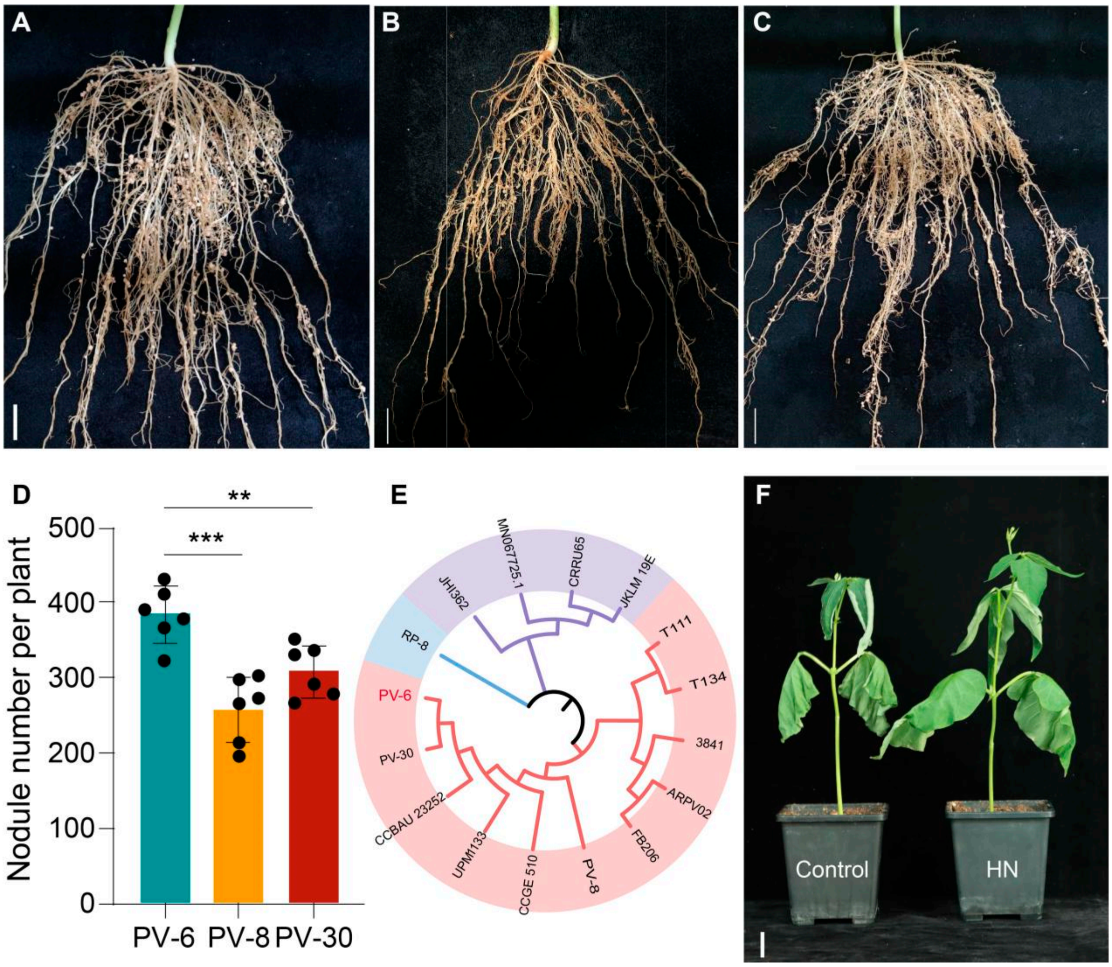

Figure S1. PV-6 strain had the best combining ability with "PinJinYun No. 3". **(A-C):** Root nodule formation phenotypes of pinJinyun 3 red kidney beans inoculated with PV-6, PV-8, and PV-30 rhizobium strains after 14 days of incubation. **(D):** Statistical analysis of nodulation counts at 14 days inoculation with PV-6, PV-8, and PV-30 rhizobium strains.The rhizobium concentration was OD_{600} = 0.06-0.08, and 30 mL of the inoculum was applied per pot. Nodulation counts were recorded for 5 randomly selected plants per treatment. Each experiment was performed with three biological replicates. Asterisks indicate statistically significant differences between rhizobial strains (*$p < 0.05$, **$p < 0.01$, ***$p < 0.001$). n.s. indicates no significant difference.Scale bar = 2 cm. **(E):** presents the phylogenetic tree of the 16S rRNA genes of PV-6, PV-8, and PV-30. The internal colors represent different branches, with the strain PV-6, highlighted in red, utilized in this experiment. **(F):** High nitrogen content significantly enhances the drought stress tolerance of red kidney beans. Seven days post-sowing, red kidney beans were subjected to a control treatment of 100 ml of distilled water and high-nitrogen treatment of 100 ml with 15.75 mmol/L KNO_3. Subsequently, a drought stress treatment was administered for a duration of seven days, where "Control" refers to the 100 ml distilled water treatment, and "HN" indicates the treatment with 100 ml of 15.75 mmol/L KNO_3. The scale bar represents 2 cm.

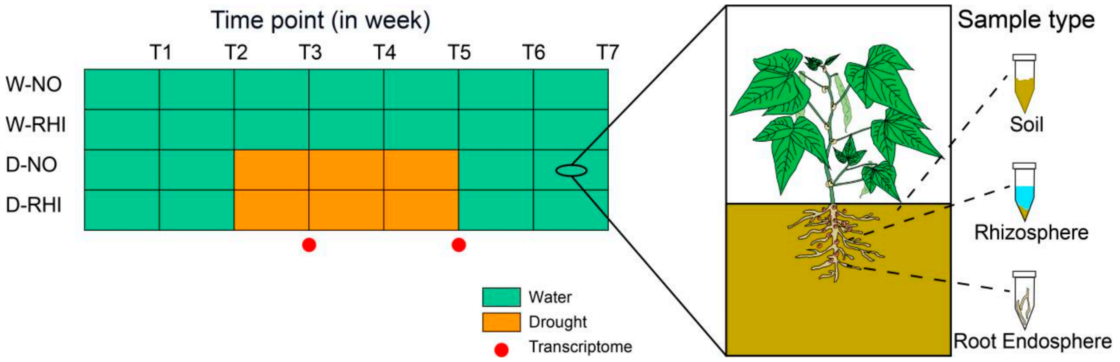

Figure S2. Red kidney bean experimental treatment diagram. Two colors represent two irrigation treatments: green indicates normal watering, and yellow indicates drought treatment. (W-NO) watering without inoculation *rhizobium*, (W-RHI) watering with inoculation *rhizobium*, (D-NO) drought without inoculation *rhizobium*, (D-RHI) drought with inoculation *rhizobium*. The samples at T1–T7 time point were sequenced by 16S amplicon, and red dots indicate transcriptome sampling time points.

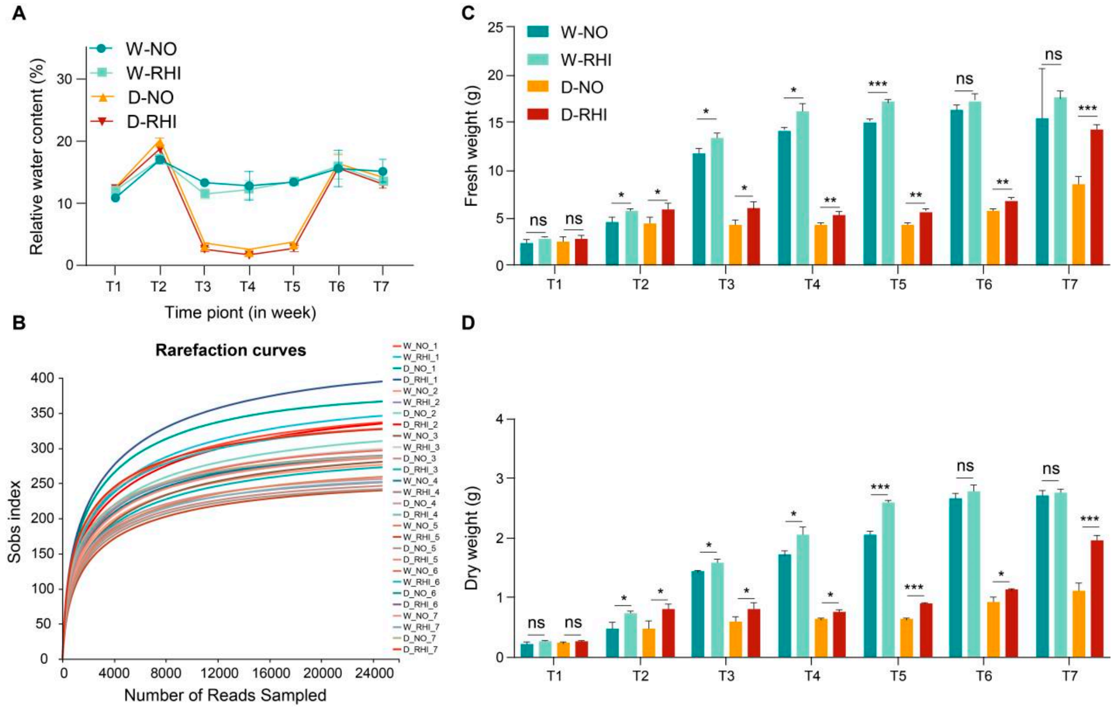

Figure S3. PV-6 enhances plant growth and drought stress tolerance of Red kidney beans. **(A):** Soil moisture content under four treatments: W-NO, W-RHI, D-NO, and D-RHI at seven time points. **(B):** Dilution curves of the four treatments: W-NO, W-RHI, D-NO, and D-RHI at seven time points. **(C-D):** Fresh weight (g) and dry weight (g) data under four treatments: W-NO, W-RHI, D-NO, and D-RHI at seven time points . Three plants were randomly selected for each treatment, and each experiment was repeated three times biologically. Asterisks indicate significant differences between the treatments (*$p < 0.05$, **$p < 0.01$, ***$p < 0.001$). n.s. indicates no significant difference.

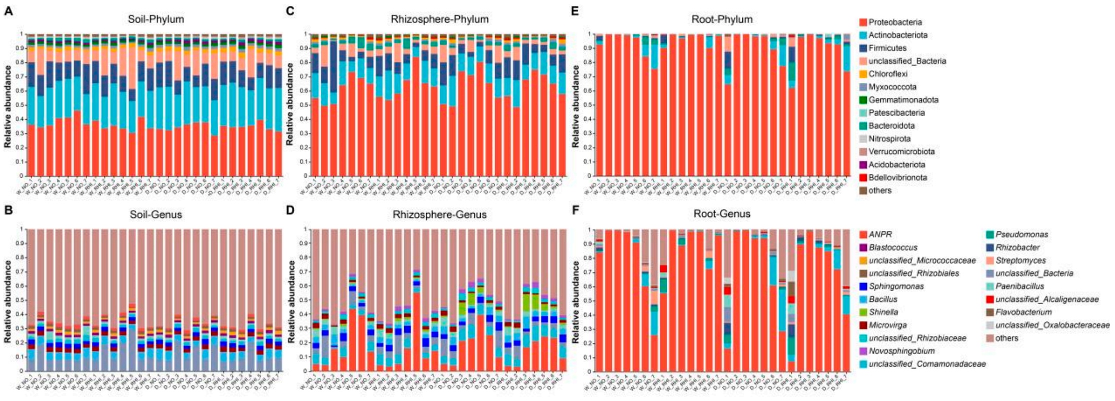

Figure S4. Microbial community of red kidney bean were changed in Phylum and Genus level. **(A-F):** Relative abundance changes in bacterial taxa at the phylum and genus levels in soil, rhizosphere, and root niches across seven time points under the four treatments: W-NO, W-RHI, D-NO, and D-RHI at seven time points. At the phylum level, bacterial phyla with an abundance proportion greater than 0.01 are displayed, while those with an abundance proportion less than 0.01 are categorized as "others". At the genus level, the top 10 bacterial genera with the highest abundance are presented, with "others" representing genera that fall below this threshold. Unresolved taxa as "unclassified"was labeled.

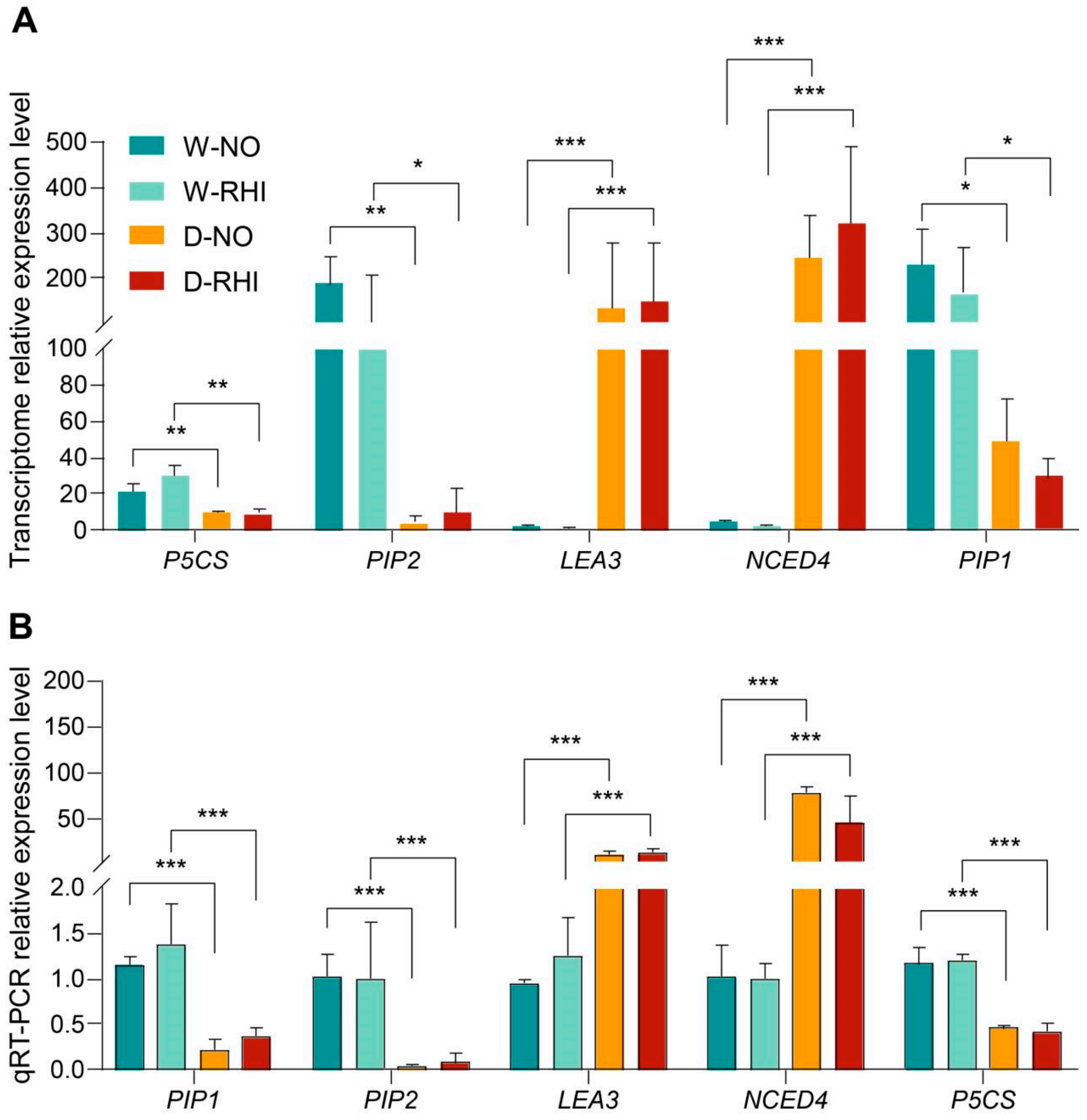

Figure S5. Drought relation marker genes expression in T5 period. **(A-B):** Transcriptome-seq and qRT-PCR analysis of marker gene expression related to drought stress at T5 stage. Three plants were randomly selected for each treatment, and each experiment was repeated three times technically. Asterisks indicate significant differences between the treatments (*$p < 0.05$, **$p < 0.01$, ***$p < 0.001$).

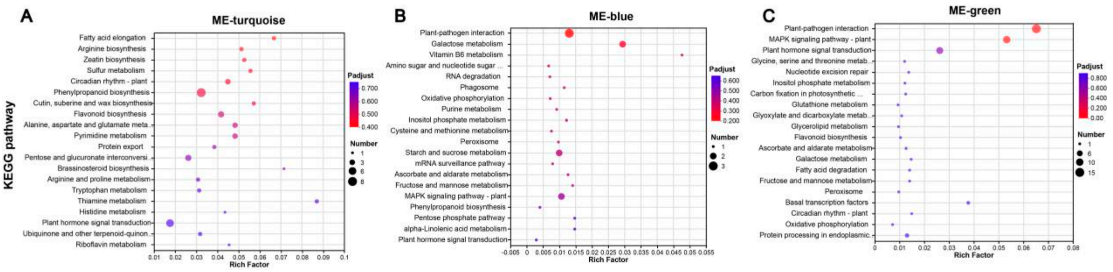

Figure S6. Modules KEGG pathway enrichment. **(A-C):** KEGG pathway enrichment analysis of gene sets from subclusters in the turquoise, blue, and green modules that met expectations.

Table S1. Amplicon sequence variants (ASVs) of 252 samples

Sample	ASV_numbers	Sample	ASV_numbers	Sample	ASV_numbers
OW_NO_e_1_1	644	OW_NO_r_1_1	3317	OW_NO_s_1_1	5067
OW_NO_e_1_2	419	OW_NO_r_1_2	3469	OW_NO_s_1_2	4789
OW_NO_e_1_3	215	OW_NO_r_1_3	3109	OW_NO_s_1_3	4574
OW_NO_e_2_1	101	OW_NO_r_2_1	4501	OW_NO_s_2_1	4595
OW_NO_e_2_2	99	OW_NO_r_2_2	3924	OW_NO_s_2_2	4494
OW_NO_e_2_3	78	OW_NO_r_2_3	3461	OW_NO_s_2_3	5092
OW_NO_e_3_1	116	OW_NO_r_3_1	1872	OW_NO_s_3_1	4865
OW_NO_e_3_2	74	OW_NO_r_3_2	1619	OW_NO_s_3_2	4448
OW_NO_e_3_3	100	OW_NO_r_3_3	1594	OW_NO_s_3_3	4370
OW_NO_e_4_1	110	OW_NO_r_4_1	3518	OW_NO_s_4_1	4175
OW_NO_e_4_2	229	OW_NO_r_4_2	2963	OW_NO_s_4_2	4147
OW_NO_e_4_3	104	OW_NO_r_4_3	2614	OW_NO_s_4_3	4254
OW_NO_e_5_1	486	OW_NO_r_5_1	889	OW_NO_s_5_1	5020
OW_NO_e_5_2	178	OW_NO_r_5_2	2658	OW_NO_s_5_2	4403
OW_NO_e_5_3	108	OW_NO_r_5_3	2085	OW_NO_s_5_3	4461
OW_NO_e_6_1	402	OW_NO_r_6_1	1887	OW_NO_s_6_1	4278
OW_NO_e_6_2	889	OW_NO_r_6_2	2433	OW_NO_s_6_2	4031
OW_NO_e_6_3	443	OW_NO_r_6_3	1365	OW_NO_s_6_3	4472
OW_NO_e_7_1	925	OW_NO_r_7_1	2785	OW_NO_s_7_1	3774
OW_NO_e_7_2	1200	OW_NO_r_7_2	2971	OW_NO_s_7_2	4179
OW_NO_e_7_3	878	OW_NO_r_7_3	2670	OW_NO_s_7_3	4219
W-NO (watering without inoculation of *rhizobium*)					
OW_RHI_e_1_1	467	OW_RHI_r_1_1	4224	OW_RHI_s_1_1	4500
OW_RHI_e_1_2	129	OW_RHI_r_1_2	3099	OW_RHI_s_1_2	4503
OW_RHI_e_1_3	713	OW_RHI_r_1_3	3440	OW_RHI_s_1_3	5107
OW_RHI_e_2_1	96	OW_RHI_r_2_1	3341	OW_RHI_s_2_1	4959
OW_RHI_e_2_2	114	OW_RHI_r_2_2	2981	OW_RHI_s_2_2	3930
OW_RHI_e_2_3	78	OW_RHI_r_2_3	3269	OW_RHI_s_2_3	4227
OW_RHI_e_3_1	245	OW_RHI_r_3_1	3595	OW_RHI_s_3_1	5180
OW_RHI_e_3_2	469	OW_RHI_r_3_2	3825	OW_RHI_s_3_2	5136
OW_RHI_e_3_3	105	OW_RHI_r_3_3	3419	OW_RHI_s_3_3	4711
OW_RHI_e_4_1	113	OW_RHI_r_4_1	2940	OW_RHI_s_4_1	4358
OW_RHI_e_4_2	120	OW_RHI_r_4_2	2878	OW_RHI_s_4_2	4354
OW_RHI_e_4_3	168	OW_RHI_r_4_3	1768	OW_RHI_s_4_3	4652
OW_RHI_e_5_1	95	OW_RHI_r_5_1	1857	OW_RHI_s_5_1	4325
OW_RHI_e_5_2	212	OW_RHI_r_5_2	1609	OW_RHI_s_5_2	4210
OW_RHI_e_5_3	77	OW_RHI_r_5_3	1752	OW_RHI_s_5_3	4190
OW_RHI_e_6_1	498	OW_RHI_r_6_1	2792	OW_RHI_s_6_1	3971
OW_RHI_e_6_2	886	OW_RHI_r_6_2	2778	OW_RHI_s_6_2	4489
OW_RHI_e_6_3	298	OW_RHI_r_6_3	3286	OW_RHI_s_6_3	4712
OW_RHI_e_7_1	155	OW_RHI_r_7_1	2738	OW_RHI_s_7_1	4673
OW_RHI_e_7_2	118	OW_RHI_r_7_2	3366	OW_RHI_s_7_2	4473

OW_RHI_e_7_3	227	OW_RHI_r_7_3	2528	OW_RHI_s_7_3	4300
W-RHI (watering with inoculation of *rhizobium*)					
D_NO_e_1_1	973	D_NO_r_1_1	3109	D_NO_s_1_1	4328
D_NO_e_1_2	1793	D_NO_r_1_2	3899	D_NO_s_1_2	4606
D_NO_e_1_3	1877	D_NO_r_1_3	2924	D_NO_s_1_3	5180
D_NO_e_2_1	180	D_NO_r_2_1	3656	D_NO_s_2_1	4601
D_NO_e_2_2	67	D_NO_r_2_2	3538	D_NO_s_2_2	4661
D_NO_e_2_3	89	D_NO_r_2_3	3695	D_NO_s_2_3	4345
D_NO_e_3_1	68	D_NO_r_3_1	2570	D_NO_s_3_1	4918
D_NO_e_3_2	110	D_NO_r_3_2	2178	D_NO_s_3_2	4569
D_NO_e_3_3	98	D_NO_r_3_3	1531	D_NO_s_3_3	4352
D_NO_e_4_1	213	D_NO_r_4_1	1281	D_NO_s_4_1	4525
D_NO_e_4_2	97	D_NO_r_4_2	1804	D_NO_s_4_2	5343
D_NO_e_4_3	377	D_NO_r_4_3	2168	D_NO_s_4_3	4542
D_NO_e_5_1	281	D_NO_r_5_1	1627	D_NO_s_5_1	4315
D_NO_e_5_2	204	D_NO_r_5_2	1363	D_NO_s_5_2	4519
D_NO_e_5_3	150	D_NO_r_5_3	1108	D_NO_s_5_3	3931
D_NO_e_6_1	464	D_NO_r_6_1	3695	D_NO_s_6_1	4057
D_NO_e_6_2	551	D_NO_r_6_2	2408	D_NO_s_6_2	3963
D_NO_e_6_3	391	D_NO_r_6_3	1647	D_NO_s_6_3	3973
D_NO_e_7_1	488	D_NO_r_7_1	2301	D_NO_s_7_1	3809
D_NO_e_7_2	780	D_NO_r_7_2	2119	D_NO_s_7_2	3212
D_NO_e_7_3	410	D_NO_r_7_3	2030	D_NO_s_7_3	6941
D-NO (drought without inoculation of *rhizobium*)					
D_RHI_e_1_1	1175	D_RHI_r_1_1	4074	D_RHI_s_1_1	4287
D_RHI_e_1_2	1373	D_RHI_r_1_2	3571	D_RHI_s_1_2	4354
D_RHI_e_1_3	1301	D_RHI_r_1_3	3424	D_RHI_s_1_3	4014
D_RHI_e_2_1	159	D_RHI_r_2_1	3990	D_RHI_s_2_1	4645
D_RHI_e_2_2	432	D_RHI_r_2_2	4152	D_RHI_s_2_2	4212
D_RHI_e_2_3	318	D_RHI_r_2_3	4508	D_RHI_s_2_3	4509
D_RHI_e_3_1	56	D_RHI_r_3_1	2139	D_RHI_s_3_1	5162
D_RHI_e_3_2	151	D_RHI_r_3_2	1430	D_RHI_s_3_2	5171
D_RHI_e_3_3	93	D_RHI_r_3_3	2242	D_RHI_s_3_3	5630
D_RHI_e_4_1	208	D_RHI_r_4_1	2508	D_RHI_s_4_1	4292
D_RHI_e_4_2	318	D_RHI_r_4_2	1772	D_RHI_s_4_2	5101
D_RHI_e_4_3	428	D_RHI_r_4_3	1187	D_RHI_s_4_3	4849
D_RHI_e_5_1	479	D_RHI_r_5_1	1908	D_RHI_s_5_1	4972
D_RHI_e_5_2	314	D_RHI_r_5_2	1679	D_RHI_s_5_2	4366
D_RHI_e_5_3	339	D_RHI_r_5_3	1904	D_RHI_s_5_3	4432
D_RHI_e_6_1	343	D_RHI_r_6_1	2198	D_RHI_s_6_1	4466
D_RHI_e_6_2	362	D_RHI_r_6_2	2309	D_RHI_s_6_2	3948
D_RHI_e_6_3	342	D_RHI_r_6_3	2511	D_RHI_s_6_3	4083
D_RHI_e_7_1	868	D_RHI_r_7_1	2578	D_RHI_s_7_1	4365
D_RHI_e_7_2	716	D_RHI_r_7_2	2678	D_RHI_s_7_2	4993

D_RHI_e_7_3	379	D_RHI_r_7_3	1716	D_RHI_s_7_3	4362

D-RHI (drought with inoculation of *rhizobium*)

Note: e: Endophytic, r: Rhizosphere, s: Soil

Table S2. Genes colone and qRT-PCR Primer sequences

Primer name	Primer sequences (5′→3′)	Usage
Actin-F	GAAGTTCTCTTCCAACCATCC	qRT-PCR
Actin-R	TTTCCTTGCTCATTCTGTCCG	qRT-PCR
PIP1-RT-F1	ACTCTCACATCCCCGTGTTG	qRT-PCR
PIP1-RT-R1	CTCCGAAGCTTCTAGCTGGG	qRT-PCR
PIP2-RT-F1	ACTCTCACATCCCCGTGTTG	qRT-PCR
PIP2-RT-R1	CTCCGAAGCTTCTAGCTGGG	qRT-PCR
LEA3-RT-F1	GGGCCGAACTGAGGAAAAGA	qRT-PCR
LEA3-RT-R1	GCCGTTTGTTGGGCCTTATC	qRT-PCR
NCED4-RT-F1	GGGAGCCTCTGTTTCTTCCC	qRT-PCR
NCED4-RT-R1	TCCACTCTTTCTCGTCGTGC	qRT-PCR
P5CS-RT-F1	AAAACGAACCGAGCTCGCAG	qRT-PCR
P5CS-RT-R1	AGAAGCCCATTCCCACTTCG	qRT-PCR
27F	AGAGTTTGATCMTGGCTCAG	Clone
1492R	GGTTACCTTGTTACGACTT	Clone

Table S3. 16s rRNA gene sequence

Strains	Sequences (5′→3′)
>PV-6	AGAGTTTGATCCTGGCTCAGAACGAACGCTGGCGGCAGGCTTAAC<br>ACATGCAAGTCGAGCGCCCCGCAAGGGGAGCGGCAGACGGGTGA<br>GTAACGCGTGGGAATCTACCCTTGACTACGGAATAACGCAGGGAA<br>ACTTGTGCTAATACCGTATGTGTCCTTCGGGAGAAAGATTTATCG<br>GTCAAGGATGAGCCCGCGTTGGATTAGCTAGTTGGTGGGGTAAAG<br>GCCTACCAAGGCGACGATCCATAGCTGGTCTGAGAGGATGATCAG<br>CCACATTGGGACTGAGACACGGCCCAAACTCCTACGGGAGGCAG<br>CAGTGGGGAATATTGGACAATGGGCGCAAGCCTGATCCAGCCATG<br>CCGCGTGAGTGATGAAGGCCCTAGGGTTGTAAAGCTCTTTCACCG<br>GAGAAGATAATGACGGTATCCGGAGAAGAAGCCCCGGCTAACTT<br>CGTGCCAGCAGCCGCGGTAATACGAAGGGGGCTAGCGTTGTTCGG<br>AATTACTGGGCGTAAAGCGCACGTAGGCGGATCGATCAGTCAGG<br>GGTGAAATCCCAGGGCTCAACCCTGGAACTGCCTTTGATACTGTC<br>GATCTGGAGTATGGAAGAGGTGAGTGGAATTCCGAGTGTAGAGG<br>TGAAATTCGTAGATATTCGGAGGAACACCAGTGGCGAAGGCGGCT<br>CACTGGTCCATTACTGACGCTGAGGTGCGAAAGCGTGGGGAGCAA<br>ACAGGATTAGATACCCTGGTAGTCCACGCCGTAAACGATGAATGT<br>TAGCCGTCGGGCAGTATACTGTTCGGTGGCGCAGCTAACGCATTA<br>AACATTCCGCCTGGGGAGTACGGTCGCAAGATTAAAACTCAAAGG<br>AATTGACGGGGGCCCGCACAAGCGGTGGAGCATGTGGTTTAATTC

	GAAGCAACGCGCAGAACCTTACCAGCCCTTGACATGCCCGGCTAC<br>TTGCAGAGATGCAAGGTTCCCTTCGGGGACCGGACACAGGTGCT<br>GCATGGCTGTCGTCAGCTCGTGTCGTGAGATGTTGGGTTAAGTCC<br>CGCAACGAGCGCAACCCTCGCCCTTAGTTGCCAGCATTCAGTTGG<br>GCACTCTAAGGGGACTGCCGGTGATAAGCCGAGAGGAAGGTGGG<br>GATGACGTCAAGTCCTCATGGCCCTTACGGGCTGGGCTACACACG<br>TGCTACAATGGTGGTGACAGTGGGCAGCGAGCACGCGAGTGTGA<br>GCTAATCTCCAAAAGCCATCTCAGTTCGGATTGCACTCTGCAACTC<br>GAGTGCATGAAGTTGGAATCGCTAGTAATCGCGGATCAGCATGCC<br>GCGGTGAATACGTTCCCGGGCCTTGTACACACCGCCCGTCACACC<br>ATGGGAGTTGGTTTTACCCGAAGGTAGTGCGCTAACCGCAAGGAG<br>GCAGCTAACCACGGTAGGGTCAGCGACTGGGGTGAAGTCGTAAC<br>AAGGTAACC
>PV-8	AGAGTTTGATCCTGGCTCAGAACGAACGCTGGCGGCAGGCTTAAC<br>ACATGCAAGTCGAACGCCCCGCAAGGGGAGTGGCAGACGGGTGA<br>GTAACGCGTGGGAACATACCCTTTCCTGCGGAATAGCTCCGGGAA<br>ACTGGAATTAATACCGCATACGCCCTACGGGGGAAAGATTTATCG<br>GGGAAGGATTGGCCCGCGTTGGATTAGCTAGTTGGTGGGGTAAAG<br>GCCTACCAAGGCGACGATCCATAGCTGGTCTGAGAGGATGATCAG<br>CCACATTGGGACTGAGACACGGCCCAAACTCCTACGGGAGGCAG<br>CAGTGGGGAATATTGGACAATGGGCGCAAGCCTGATCCAGCCATG<br>CCGCGTGAGTGATGAAGGCCTTAGGGTTGTAAAGCTCTTTCACCG<br>GAGAAGATAATGACGGTATCCGGAGAAGAAGCCCCGGCTAACTT<br>CGTGCCAGCAGCCGCGGTAATACGAAGGGGGCTAGCGTTGTTCGG<br>AATTACTGGGCGTAAAGCGCACGTAGGCGGATATTTAAGTCAGGG<br>GTGAAATCCCAGAGCTCAACTCTGGAACTGCCTTTGATACTGGGT<br>ATCTTGAGTATGGAAGAGGTAAGTGGAATTCCGAGTGTAGAGGTG<br>AAATTCGTAGATATTCGGAGGAACACCAGTGGCGAAGGCGGCTTA<br>CTGGTCCATTACTGACGCTGAGGTGCGAAAGCGTGGGGAGCAAAC<br>AGGATTAGATACCCTGGTAGTCCACGCCGTAAACGATGAATGTTA<br>GCCGTCGGGCAGTATACTGTTCGGTGGCGCAGCTAACGCATTAAA<br>CATTCCGCCTGGGGAGTACGGTCGCAAGATTAAAACTCAAAGGAA<br>TTGACGGGGGCCCGCACAAGCGGTGGAGCATGTGGTTTAATTCGA<br>AGCAACGCGCAGAACCTTACCAGCTCTTGACATTCGGGGTATGGG<br>CATTGGAGACGATGTCCTTCAGTTAGGCTGGCCCCAGAACAGGTG<br>CTGCATGGCTGTCGTCAGCTCGTGTCGTGAGATGTTGGGTTAAGTC<br>CCGCAACGAGCGCAACCCTCGCCCTTAGTTGCCAGCATTCAGTTG<br>GGCACTCTAAGGGGACTGCCGGTGATAAGCCGAGAGGAAGGTGG<br>GGATGACGTCAAGTCCTCATGGCCCTTACGGGCTGGGCTACACAC<br>GTGCTACAATGGTGGTGACAGTGGGCAGCGAGACAGCGATGTCG<br>AGCTAATCTCCAAAAGCCATCTCAGTTCGGATTGCACTCTGCAAC<br>TCGAGTGCATGAAGTTGGAATCGCTAGTAATCGCAGATCAGCATG<br>CTGCGGTGAATACGTTCCCGGGCCTTGTACACACCGCCCGTCACA<br>CCATGGGAGTTGGTTTTACCCGAAGGTAGTGCGCTAACCGCAAGG

	AGGCAGCTAACCACGGTAGGGTCAGCGACTGGGGTGAAGTCGTA<br>ACAAGGTAACC
>PV-30	AGAGTTTGATCCTGGCTCAGAACGAACGCTGGCGGCAGGCTTAAC<br>ACATGCAAGTCGAGCGCCCCGCAAGGGGAGCGGCAGACGGGTGA<br>GTAACGCGTGGGAATCTACCCTTGACTACGGAATAACGCAGGGAA<br>ACTTGTGCTAATACCGTATGTGTCCTTCGGGAGAAAGATTTATCG<br>GTCAAGGATGAGCCCGCGTTGGATTAGCTAGTTGGTGGGGTAAAG<br>GCCTACCAAGGCGACGATCCATAGCTGGTCTGAGAGGATGATCAG<br>CCACATTGGGACTGAGACACGGCCCAAACTCCTACGGGAGGCAG<br>CAGTGGGGAATATTGGACAATGGGCGCAAGCCTGATCCAGCCATG<br>CCGCGTGAGTGATGAAGGCCCTAGGGTTGTAAAGCTCTTTCACCG<br>GAGAAGATAATGACGGTATCCGGAGAAGAAGCCCCGGCTAACTT<br>CGTGCCAGCAGCCGCGGTAATACGAAGGGGGCTAGCGTTGTTCGG<br>AATTACTGGGCGTAAAGCGCACGTAGGCGGATCGATCAGTCAGG<br>GGTGAAATCCCAGGGCTCAACCCTGGAACTGCCTTTGATACTGTC<br>GATCTGGAGTATGGAAGAGGTGAGTGGAATTCCGAGTGTAGAGG<br>TGAAATTCGTAGATATTCGGAGGAACACCAGTGGCGAAGGCGGCT<br>CACTGGTCCATTACTGACGCTGAGGTGCGAAAGCGTGGGGAGCAA<br>ACAGGATTAGATACCCTGGTAGTCCACGCCGTAAACGATGAATGT<br>TAGCCGTCGGGCAGTATACTGTTCGGTGGCGCAGCTAACGCATTA<br>AACATTCCGCCTGGGGAGTACGGTCGCAAGATTAAAACTCAAAGG<br>AATTGACGGGGGCCCGCACAAGCGGTGGAGCATGTGGTTTAATTC<br>GAAGCAACGCGCAGAACCTTACCAGCCCTTGACATGCCCGGCTAC<br>TTGCAGAGATGCAAGGTTCCCTTCGGGGACCGGGACACAGGTGCT<br>GCATGGCTGTCGTCAGCTCGTGTCGTGAGATGTTGGGTTAAGTCC<br>CGCAACGAGCGCAACCCTCGCCCTTAGTTGCCAGCATTTGGTTGG<br>GCACTCTAAGGGGACTGCCGGTGATAAGCCGAGAGGAAGGTGGG<br>GATGACGTCAAGTCCTCATGGCCCTTACGGGCTGGGCTACACACG<br>TGCTACAATGGTGGTGACAGTGGGCAGCGAGCACGCGAGTGTGA<br>GCTAATCTCCAAAAGCCATCTCAGTTCGGATTGCACTCTGCAACTC<br>GAGTGCATGAAGTTGGAATCGCTAGTAATCGCGGATCAGCATGCC<br>GCGGTGAATACGTTCCCGGGCCTTGTACACACCGCCCGTCACACC<br>ATGGGAGTTGGTTTTACCCGAAGGTAGTGCGCTAACCGCAAGGAG<br>GCAGCTAACCACGGTAGGGTCAGCGACTGGGGTGAAGTCGTAAC<br>AAGGTAACC
>CCBAU 23252	AGAGTTTGATCCTGGCTCAGAACGAACGCTGGCGGCAGGCTTAAC<br>ACATGCAAGTCGAGCGCCCCGCAAGGGGAGCGGCAGACGGGTGA<br>GTAACGCGTGGGAATCTACCCTTGACTACGGAATAACGCAGGGAA<br>ACTTGTGCTAATACCGTATGTGTCCTTCGGGAGAAAGATTTATCG<br>GTCAAGGATGAGCCCGCGTTGGATTAGCTAGTTGGTGGGGTAAAG<br>GCCTACCAAGGCGACGATCCATAGCTGGTCTGAGAGGATGATCAG<br>CCACATTGGGACTGAGACACGGCCCAAACTCCTACGGGAGGCAG<br>CAGTGGGGAATATTGGACAATGGGCGCAAGCCTGATCCAGCCATG<br>CCGCGTGAGTGATGAAGGCCCTAGGGTTGTAAAGCTCTTTCACCG

	GAGAAGATAATGACGGTATCCGGAGAAGAAGCCCCGGCTAACTT<br>CGTGCCAGCAGCCGCGGTAATACGAAGGGGGCTAGCGTTGTTCGG<br>AATTACTGGGCGTAAAGCGCACGTAGGCGGATCGATCAGTCAGG<br>GGTGAAATCCCAGGGCTCAACCCTGGAACTGCCTTTGATACTGTC<br>GATCTGGAGTATGGAAGAGGTGAGTGGAATTCCGAGTGTAGAGG<br>TGAAATTCGTAGATATTCGGAGGAACACCAGTGGCGAAGGCGGCT<br>CACTGGTCCATTACTGACGCTGAGGTGCGAAAGCGTGGGGAGCAA<br>ACAGGATTAGATACCCTGGTAGTCCACGCCGTAAACGATGAATGT<br>TAGCCGTCGGGCAGTATACTGTTCGGTGGCGCAGCTAACGCATTA<br>AACATTCCGCCTGGGGAGTACGGTCGCAAGATTAAAACTCAAAGG<br>AATTGACGGGGGCCCGCACAAGCGGTGGAGCATGTGGTTTAATTC<br>GAAGCAACGCGCAGAACCTTACCAGCCCTTGACATGCCCGGCTAC<br>TTGCAGAGATGCAAGGTTCCCTTCGGGGACCGGGACACAGGTGCT<br>GCATGGCTGTCGTCAGCTCGTGTCGTGAGATGTTGGGTTAAGTCC<br>CGCAACGAGCGCAACCCTCGCCCTTAGTTGCCAGCATTCAGTTGG<br>GCACTCTAAGGGGACTGCCGGTGATAAGCCGAGAGGAAGGTGGG<br>GATGACGTCAAGTCCTCATGGCCCTTACGGGCTGGGCTACACACG<br>TGCTACAATGGTGGTGACAGTGGGCAGCGAGCACGCGAGTGTGA<br>GCTAATCTCCAAAAGCCATCTCAGTTCGGATTGCACTCTGCAACTC<br>GAGTGCATGAAGTTGGAATCGCTAGTAATCGCGGATCAGCATGCC<br>GCGGTGAATACGTTCCCGGGCCTTGTACACACCGCCCGTCACACC<br>ATGGGAGTTGGTTTTACCCGAAGGTAGTGCGCTAACCGCAAGGAG<br>GCAGCTAACCACGGTAGGGTCAGCGACTGGGGTGAAGTCGTAAC<br>AAGGTAGCCGTAGGGGAACCTGCGGCTGGATCACCT
>UPM1133	AGAGTTTGATCCTGGCTCAGAACGAACGCTGGCGGCAGGCTTAAC<br>ACATGCAAGTCGAGCGCCCCGCAAGGGGAGCGGCAGACGGGTGA<br>GTAACGCGTGGGAATCTACCCTTGACTACGGAATAACGCAGGGAA<br>ACTTGTGCTAATACCGTATGTGTCCTTCGGGAGAAAGATTTATCG<br>GTCAAGGATGAGCCCGCGTTGGATTAGCTAGTTGGTGGGGTAAAG<br>GCCTACCAAGGCGACGATCCATAGCTGGTCTGAGAGGATGATCAG<br>CCACATTGGGACTGAGACACGGCCCAAACTCCTACGGGAGGCAG<br>CAGTGGGGAATATTGGACAATGGGCGCAAGCCTGATCCAGCCATG<br>CCGCGTGAGTGATGAAGGCCCTAGGGTTGTAAAGCTCTTTCACCG<br>GAGAAGATAATGACGGTATCCGGAGAAGAAGCCCCGGCTAACTT<br>CGTGCCAGCAGCCGCGGTAATACGAAGGGGGCTAGCGTTGTTCGG<br>AATTACTGGGCGTAAAGCGCACGTAGGCGGATCGATCAGTCAGG<br>GGTGAAATCCCAGGGCTCAACCCTGGAACTGCCTTTGATACTGTC<br>GATCTGGAGTATGGAAGAGGTGAGTGGAATTCCGAGTGTAGAGG<br>TGAAATTCGTAGATATTCGGAGGAACACCAGTGGCGAAGGCGGCT<br>CACTGGTCCATTACTGACGCTGAGGTGCGAAAGCGTGGGGAGCAA<br>ACAGGATTAGATACCCTGGTAGTCCACGCCGTAAACGATGAATGT<br>TAGCCGTCGGGCAGTATACTGTTCGGTGGCGCAGCTAACGCATTA<br>AACATTCCGCCTGGGGAGTACGGTCGCAAGATTAAAACTCAAAGG<br>AATTGACGGGGGCCCGCACAAGCGGTGGAGCATGTGGTTTAATTC

	GAAGCAACGCGCAGAACCTTACCAGCCCTTGACATGCCCGGCTAC<br>TTGCAGAGATGCAAGGTTCCCTTCGGGGACCGGGACACAGGTGCT<br>GCATGGCTGTCGTCAGCTCGTGTCGTGAGATGTTGGGTTAAGTCC<br>CGCAACGAGCGCAACCCTCGCCCTTAGTTGCCAGCATTCAGTTGG<br>GCACTCTAAGGGGACTGCCGGTGATAAGCCGAGAGGAAGGTGGG<br>GATGACGTCAAGTCCTCATGGCCCTTACGGGCTGGGCTACACACG<br>TGCTACAATGGTGGTGACAGTGGGCAGCGAGCACGCGAGTGTGA<br>GCTAATCTCCAAAAGCCATCTCAGTTCGGATTGCACTCTGCAACTC<br>GAGTGCATGAAGTTGGAATCGCTAGTAATCGCGGATCAGCATGCC<br>GCGGTGAATACGTTCCCGGGCCTTGTACACACCGCCCGTCACACC<br>ATGGGAGTTGGTTTTACCCGAAGGTAGTGCGCTAACCGCAAGGAG<br>GCAGCTAACCACGGTAGGGTCAGCGACTGGGGTGAAGTCGTAAC<br>AAGGTAGCCGTAGGGGAACCTGCGGCTGGATCACCT
>JKLM 19E	TCTCAACATGAGAGTTTGATCCTGGCTCAGAACGAACGCTGGCGG<br>CAGGCTTAACACATGCAAGTCGAGCGCCCCGCAAGGGGAGCGGC<br>AGACGGGTGAGTAACGCGTGGGAATCTACCCTTGACTACGGAATA<br>ACGCAGGGAAACTTGTGCTAATACCGTATGTGTCCTTCGGGAGAA<br>AGATTTATCGGTCAAGGATGAGCCCGCGTTGGATTAGCTAGTTGG<br>TGGGGTAAAGGCCTACCAAGGCGACGATCCATAGCTGGTCTGAGA<br>GGATGATCAGCCACATTGGGACTGAGACACGGCCCAAACTCCTAC<br>GGGAGGCAGCAGTGGGGAATATTGGACAATGGGCGCAAGCCTGA<br>TCCAGCCATGCCGCGTGAGTGATGAAGGCCCTAGGGTTGTAAAGC<br>TCTTTCACCGGAGAAGATAATGACGGTATCCGGAGAAGAAGCCCC<br>GGCTAACTTCGTGCCAGCAGCCGCGGTAATACGAAGGGGGCTAGC<br>GTTGTTCGGAATTACTGGGCGTAAAGCGCACGTAGGCGGATCGAT<br>CAGTCAGGGGTGAAATCCCAGGGCTCAACCCTGGAACTGCCTTTG<br>ATACTGTCGATCTGGAGTATGGAAGAGGTGAGTGGAATTCCGAGT<br>GTAGAGGTGAAATTCGTAGATATTCGGAGGAACACCAGTGGCGA<br>AGGCGGCTCACTGGTCCATTACTGACGCTGAGGTGCGAAAGCGTG<br>GGGAGCAAACAGGATTAGATACCCTGGTAGTCCACGCCGTAAAC<br>GATGAATGTTAGCCGTCGGGCAGTATACTGTTCGGTGGCGCAGCT<br>AACGCATTAAACATTCCGCCTGGGGAGTACGGTCGCAAGATTAAA<br>ACTCAAAGGAATTGACGGGGGCCCGCACAAGCGGTGGAGCATGT<br>GGTTTAATTCGAAGCAACGCGCAGAACCTTACCAGCCCTTGACAT<br>GCCCGGCTACTTGCAGAGATGCAAGGTTCCCTTCGGGGACCGGGA<br>CACAGGTGCTGCATGGCTGTCGTCAGCTCGTGTCGTGAGATGTTG<br>GGTTAAGTCCCGCAACGAGCGCAACCCTCGCCCTTAGTTGCCAGC<br>ATTCAGTTGGGCACTCTAAGGGGACTGCCGGTGATAAGCCGAGAG<br>GAAGGTGGGGATGACGTCAAGTCCTCATGGCCCTTACGGGCTGGG<br>CTACACACGTGCTACAATGGTGGTGACAGTGGGCAGCGAGCACGC<br>GAGTGTGAGCTAATCTCCAAAAGCCATCTCAGTTCGGATTGCACT<br>CTGCAACTCGAGTGCATGAAGTTGGAATCGCTAGTAATCGCGGAT<br>CAGCATGCCGCGGTGAATACGTTCCCGGGCCTTGTACACACCGCC<br>CGTCACACCATGGGAGTTGGTTTTACCCGAAGGTAGTGCGCTAAC

	CGCAAGGAGGCAGCTAACCACGGTAGGGTCAGCGACTGGGGTGA<br>AGTCGTAACAAGGTAGCCGTAGGGGAACCTGCGGCTGGATCACCT<br>CCTTTCTA
>CRRU65	TCTCAACATGAGAGTTTGATCCTGGCTCAGAACGAACGCTGGCGG<br>CAGGCTTAACACATGCAAGTCGAGCGCCCCGCAAGGGGAGCGGC<br>AGACGGGTGAGTAACGCGTGGGAATCTACCCTTGACTACGGAATA<br>ACGCAGGGAAACTTGTGCTAATACCGTATGTGTCCTTCGGGAGAA<br>AGATTTATCGGTCAAGGATGAGCCCGCGTTGGATTAGCTAGTTGG<br>TGGGGTAAAGGCCTACCAAGGCGACGATCCATAGCTGGTCTGAGA<br>GGATGATCAGCCACATTGGGACTGAGACACGGCCCAAACTCCTAC<br>GGGAGGCAGCAGTGGGGAATATTGGACAATGGGCGCAAGCCTGA<br>TCCAGCCATGCCGCGTGAGTGATGAAGGCCCTAGGGTTGTAAAGC<br>TCTTTCACCGGAGAAGATAATGACGGTATCCGGAGAAGAAGCCCC<br>GGCTAACTTCGTGCCAGCAGCCGCGGTAATACGAAGGGGGCTAGC<br>GTTGTTCGGAATTACTGGGCGTAAAGCGCACGTAGGCGGATCGAT<br>CAGTCAGGGGTGAAATCCCAGGGCTCAACCCTGGAACTGCCTTTG<br>ATACTGTCGATCTGGAGTATGGAAGAGGTGAGTGGAATTCCGAGT<br>GTAGAGGTGAAATTCGTAGATATTCGGAGGAACACCAGTGGCGA<br>AGGCGGCTCACTGGTCCATTACTGACGCTGAGGTGCGAAAGCGTG<br>GGGAGCAAACAGGATTAGATACCCTGGTAGTCCACGCCGTAAAC<br>GATGAATGTTAGCCGTCGGGCAGTATACTGTTCGGTGGCGCAGCT<br>AACGCATTAAACATTCCGCCTGGGGAGTACGGTCGCAAGATTAAA<br>ACTCAAAGGAATTGACGGGGGCCCGCACAAGCGGTGGAGCATGT<br>GGTTTAATTCGAAGCAACGCGCAGAACCTTACCAGCCCTTGACAT<br>GCCCGGCTACTTGCAGAGATGCAAGGTTCCCTTCGGGGACCGGGA<br>CACAGGTGCTGCATGGCTGTCGTCAGCTCGTGTCGTGAGATGTTG<br>GGTTAAGTCCCGCAACGAGCGCAACCCTCGCCCTTAGTTGCCAGC<br>ATTCAGTTGGGCACTCTAAGGGGACTGCCGGTGATAAGCCGAGAG<br>GAAGGTGGGGATGACGTCAAGTCCTCATGGCCCTTACGGGCTGGG<br>CTACACACGTGCTACAATGGTGGTGACAGTGGGCAGCGAGCACGC<br>GAGTGTGAGCTAATCTCCAAAAGCCATCTCAGTTCGGATTGCACT<br>CTGCAACTCGAGTGCATGAAGTTGGAATCGCTAGTAATCGCGGAT<br>CAGCATGCCGCGGTGAATACGTTCCCGGGCCTTGTACACACCGCC<br>CGTCACACCATGGGAGTTGGTTTTACCCGAAGGTAGTGCGCTAAC<br>CGCAAGGAGGCAGCTAACCACGGTAGGGTCAGCGACTGGGGTGA<br>AGTCGTAACAAGGTAGCCGTAGGGGAACCTGCGGCTGGATCACCT<br>CCTTTCTA
>CCGE 510	AGAGTTTGATCCTGGCTCAGAACGAACGCTGGCGGCAGGCTTAAC<br>ACATGCAAGTCGAGCGCCCCGCAAGGGGAGCGGCAGACGGGTGA<br>GTAACGCGTGGGAATCTACCCTTGACTACGGAATAACGCAGGGAA<br>ACTTGTGCTAATACCGTATGTGTCCTTCGGGAGAAAGATTTATCG<br>GTCAAGGATGAGCCCGCGTTGGATTAGCTAGTTGGTGGGGTAAAG<br>GCCTACCAAGGCGACGATCCATAGCTGGTCTGAGAGGATGATCAG<br>CCACATTGGGACTGAGACACGGCCCAAACTCCTACGGGAGGCAG

	CAGTGGGGAATATTGGACAATGGGCGCAAGCCTGATCCAGCCATG<br>CCGCGTGAGTGATGAAGGCCCTAGGGTTGTAAAGCTCTTTCACCG<br>GAGAAGATAATGACGGTATCCGGAGAAGAAGCCCCGGCTAACTT<br>CGTGCCAGCAGCCGCGGTAATACGAAGGGGGCTAGCGTTGTTCGG<br>AATTACTGGGCGTAAAGCGCACGTAGGCGGATCGATCAGTCAGG<br>GGTGAAATCCCAGGGCTCAACCCTGGAACTGCCTTTGATACTGTC<br>GATCTGGAGTATGGAAGAGGTGAGTGGAATTCCGAGTGTAGAGG<br>TGAAATTCGTAGATATTCGGAGGAACACCAGTGGCGAAGGCGGCT<br>CACTGGTCCATTACTGACGCTGAGGTGCGAAAGCGTGGGGAGCAA<br>ACAGGATTAGATACCCTGGTAGTCCACGCCGTAAACGATGAATGT<br>TAGCCGTCGGGCAGTATACTGTTCGGTGGCGCAGCTAACGCATTA<br>AACATTCCGCCTGGGGAGTACGGTCGCAAGATTAAAACTCAAAGG<br>AATTGACGGGGGCCCGCACAAGCGGTGGAGCATGTGGTTTAATTC<br>GAAGCAACGCGCAGAACCTTACCAGCCCTTGACATGCCCGGCTAC<br>TTGCAGAGATGCAAGGTTCCCTTCGGGGACCGGGACACAGGTGCT<br>GCATGGCTGTCGTCAGCTCGTGTCGTGAGATGTTGGGTTAAGTCC<br>CGCAACGAGCGCAACCCTCGCCCTTAGTTGCCAGCATTCAGTTGG<br>GCACTCTAAGGGGACTGCCGGTGATAAGCCGAGAGGAAGGTGGG<br>GATGACGTCAAGTCCTCATGGCCCTTACGGGCTGGGCTACACACG<br>TGCTACAATGGTGGTGACAGTGGGCAGCGAGCACGCGAGTGTGA<br>GCTAATCTCCAAAAGCCATCTCAGTTCGGATTGCACTCTGCAACTC<br>GAGTGCATGAAGTTGGAATCGCTAGTAATCGCGGATCAGCATGCC<br>GCGGTGAATACGTTCCCGGGCCTTGTACACACCGCCCGTCACACC<br>ATGGGAGTTGGTTTTACCCGAAGGTAGTGCGCTAACCGCAAGGAG<br>GCAGCTAACCACGGTAGGGTCAGCGACTGGGGTGAAGTCGTAAC<br>AAGGTAGCCGTAGGGGAACCTGCGGCTGGATCACCTCCTT
>JHI362	ATGAGAGTTTGATCCTGGCTCAGAACGAACGCTGGCGGCAGGCTT<br>AACACATGCAAGTCGAGCGCCCCGCAAGGGGAGCGGCAGACGGG<br>TGAGTAACGCGTGGGAATCTACCCTTGACTACGGAATAACGCAGG<br>GAAACTTGTGCTAATACCGTATGTGTCCTTCGGGAGAAAGATTTA<br>TCGGTCAAGGATGAGCCCGCGTTGGATTAGCTAGTTGGTGGGGTA<br>AAGGCCTACCAAGGCGACGATCCATAGCTGGTCTGAGAGGATGAT<br>CAGCCACATTGGGACTGAGACACGGCCCAAACTCCTACGGGAGG<br>CAGCAGTGGGGAATATTGGACAATGGGCGCAAGCCTGATCCAGC<br>CATGCCGCGTGAGTGATGAAGGCCCTAGGGTTGTAAAGCTCTTTC<br>ACCGGAGAAGATAATGACGGTATCCGGAGAAGAAGCCCCGGCTA<br>ACTTCGTGCCAGCAGCCGCGGTAATACGAAGGGGGCTAGCGTTGT<br>TCGGAATTACTGGGCGTAAAGCGCACGTAGGCGGATCGATCAGTC<br>AGGGGTGAAATCCCAGGGCTCAACCCTGGAACTGCCTTTGATACT<br>GTCGATCTGGAGTATGGAAGAGGTGAGTGGAATTCCGAGTGTAGA<br>GGTGAAATTCGTAGATATTCGGAGGAACACCAGTGGCGAAGGCG<br>GCTCACTGGTCCATTACTGACGCTGAGGTGCGAAAGCGTGGGGAG<br>CAAACAGGATTAGATACCCTGGTAGTCCACGCCGTAAACGATGAA<br>TGTTAGCCGTCGGGCAGTATACTGTTCGGTGGCGCAGCTAACGCA

	TTAAACATTCCGCCTGGGGAGTACGGTCGCAAGATTAAAACTCAA<br>AGGAATTGACGGGGGCCCGCACAAGCGGTGGAGCATGTGGTTTA<br>ATTCGAAGCAACGCGCAGAACCTTACCAGCCCTTGACATGCCCGG<br>CTACTTGCAGAGATGCAAGGTTCCCTTCGGGGACCGGACACAGG<br>TGCTGCATGGCTGTCGTCAGCTCGTGTCGTGAGATGTTGGGTTAA<br>GTCCCGCAACGAGCGCAACCCTCGCCCTTAGTTGCCAGCATTCAG<br>TTGGGCACTCTAAGGGGACTGCCGGTGATAAGCCGAGAGGAAGG<br>TGGGGATGACGTCAAGTCCTCATGGCCCTTACGGGCTGGGCTACA<br>CACGTGCTACAATGGTGGTGACAGTGGGCAGCGAGCACGCGAGT<br>GTGAGCTAATCTCCAAAAGCCATCTCAGTTCGGATTGCACTCTGC<br>AACTCGAGTGCATGAAGTTGGAATCGCTAGTAATCGCGGATCAGC<br>ATGCCGCGGTGAATACGTTCCCGGGCCTTGTACACACCGCCCGTC<br>ACACCATGGGAGTTGGTTTTACCCGAAGGTAGTGCGCTAACCGCA<br>AGGAGGCAGCTAACCACGGTAGGGTCAGCGACTGGGGTGAAGTC<br>GTAACAAGGTAGCCGTAGGGGAACCTGCGGCTGGATCACCTCCTT<br>T
>FB206	TAGAGTTTGATCCTGGCTCAGAACGAACGCTGGCGGCAGGCTTAA<br>CACATGCAAGTCGAGCGCCCCGCAAGGGGAGCGGCAGACGGGTG<br>AGTAACGCGTGGGAATCTACCCTTGACTACGGAATAACGCAGGGA<br>AACTTGTGCTAATACCGTATGTGTCCTTCGGGAGAAAGATTTATC<br>GGTCAAGGATGAGCCCGCGTTGGATTAGCTAGTTGGTGGGGTAAA<br>GGCCTACCAAGGCGACGATCCATAGCTGGTCTGAGAGGATGATCA<br>GCCACATTGGGACTGAGACACGGCCCAAACTCCTACGGGAGGCA<br>GCAGTGGGGAATATTGGACAATGGGCGCAAGCCTGATCCAGCCAT<br>GCCGCGTGAGTGATGAAGGCCCTAGGGTTGTAAAGCTCTTTCACC<br>GGAGAAGATAATGACGGTATCCGGAGAAGAAGCCCCGGCTAACT<br>TCGTGCCAGCAGCCGCGGTAATACGAAGGGGGCTAGCGTTGTTCG<br>GAATTACTGGGCGTAAAGCGCACGTAGGCGGATCGATCAGTCAG<br>GGGTGAAATCCCAGGGCTCAACCCTGGAACTGCCTTTGATACTGT<br>CGATCTGGAGTATGGAAGAGGTGAGTGGAATTCCGAGTGTAGAG<br>GTGAAATTCGTAGATATTCGGAGGAACACCAGTGGCGAAGGCGG<br>CTCACTGGTCCATTACTGACGCTGAGGTGCGAAAGCGTGGGGAGC<br>AAACAGGATTAGATACCCTGGTAGTCCACGCCGTAAACGATGAAT<br>GTTAGCCGTCGGGCAGTATACTGTTCGGTGGCGCAGCTAACGCAT<br>TAAACATTCCGCCTGGGGAGTACGGTCGCAAGATTAAAACTCAAA<br>GGAATTGACGGGGGCCCGCACAAGCGGTGGAGCATGTGGTTTAAT<br>TCGAAGCAACGCGCAGAACCTTACCAGCCCTTGACATGCCCGGCT<br>ACTTGCAGAGATGCAAGGTTCCCTTCGGGGACCGGACACAGGTG<br>CTGCATGGCTGTCGTCAGCTCGTGTCGTGAGATGTTGGGTTAAGTC<br>CCGCAACGAGCGCAACCCTCGCCCTTAGTTGCCAGCATTCAGTTG<br>GGCACTCTAAGGGGACTGCCGGTGATAAGCCGAGAGGAAGGTGG<br>GGATGACGTCAAGTCCTCATGGCCCTTACGGGCTGGGCTACACAC<br>GTGCTACAATGGTGGTGACAGTGGGCAGCGAGCACGCGAGTGTG<br>AGCTAATCTCCAAAAGCCATCTCAGTTCGGATTGCACTCTGCAAC

	TCGAGTGCATGAAGTTGGAATCGCTAGTAATCGCGGATCAGCATG<br>CCGCGGTGAATACGTTCCCGGGCCTTGTACACACCGCCCGTCACA<br>CCATGGGAGTTGGTTTTACCCGAAGGTAGTGCGCTAACCGCAAGG<br>AGGCAGCTAACCACGGTAGGGTCAGCGACTGGGGTGAAGTCGTA<br>ACAAGGTAGCCGTAGGGGAACCTGCGGCTGGATCACCTCCTTA
>3841	CAACATGAGAGTTTGATCCTGGCTCAGAACGAACGCTGGCGGCAG<br>GCTTAACACATGCAAGTCGAGCGCCCCGCAAGGGGAGCGGCAGA<br>CGGGTGAGTAACGCGTGGGAATCTACCCTTGACTACGGAATAACG<br>CAGGGAAACTTGTGCTAATACCGTATGTGTCCTTCGGGAGAAAGA<br>TTTATCGGTCAAGGATGAGCCCGCGTTGGATTAGCTAGTTGGTGG<br>GGTAAAGGCCTACCAAGGCGACGATCCATAGCTGGTCTGAGAGG<br>ATGATCAGCCACATTGGGACTGAGACACGGCCCAAACTCCTACGG<br>GAGGCAGCAGTGGGGAATATTGGACAATGGGCGCAAGCCTGATC<br>CAGCCATGCCGCGTGAGTGATGAAGGCCCTAGGGTTGTAAAGCTC<br>TTTCACCGGAGAAGATAATGACGGTATCCGGAGAAGAAGCCCCG<br>GCTAACTTCGTGCCAGCAGCCGCGGTAATACGAAGGGGGCTAGCG<br>TTGTTCGGAATTACTGGGCGTAAAGCGCACGTAGGCGGATCGATC<br>AGTCAGGGGTGAAATCCCAGGGCTCAACCCTGGAACTGCCTTTGA<br>TACTGTCGATCTGGAGTATGGAAGAGGTGAGTGGAATTCCGAGTG<br>TAGAGGTGAAATTCGTAGATATTCGGAGGAACACCAGTGGCGAA<br>GGCGGCTCACTGGTCCATTACTGACGCTGAGGTGCGAAAGCGTGG<br>GGAGCAAACAGGATTAGATACCCTGGTAGTCCACGCCGTAAACG<br>ATGAATGTTAGCCGTCGGGCAGTATACTGTTCGGTGGCGCAGCTA<br>ACGCATTAAACATTCCGCCTGGGGAGTACGGTCGCAAGATTAAAA<br>CTCAAAGGAATTGACGGGGGCCCGCACAAGCGGTGGAGCATGTG<br>GTTTAATTCGAAGCAACGCGCAGAACCTTACCAGCCCTTGACATG<br>CCCGGCTACTTGCAGAGATGCAAGGTTCCCTTCGGGGACCGGGAC<br>ACAGGTGCTGCATGGCTGTCGTCAGCTCGTGTCGTGAGATGTTGG<br>GTTAAGTCCCGCAACGAGCGCAACCCTCGCCCTTAGTTGCCAGCA<br>TTTAGTTGGGCACTCTAAGGGGACTGCCGGTGATAAGCCGAGAGG<br>AAGGTGGGGATGACGTCAAGTCCTCATGGCCCTTACGGGCTGGGC<br>TACACACGTGCTACAATGGTGGTGACAGTGGGCAGCGAGCACGC<br>GAGTGTGAGCTAATCTCCAAAAGCCATCTCAGTTCGGATTGCACT<br>CTGCAACTCGAGTGCATGAAGTTGGAATCGCTAGTAATCGCGGAT<br>CAGCATGCCGCGGTGAATACGTTCCCGGGCCTTGTACACACCGCC<br>CGTCACACCATGGGAGTTGGTTTTACCCGAAGGTAGTGCGCTAAC<br>CGCAAGGAGGCAGCTAACCACGGTAGGGTCAGCGACTGGGGTGA<br>AGTCGTAACAAGGTAGCCGTAGGGGAACCTGCGGCTGGATCACCT<br>CCTTT
>ARPV02	TAGAGTTTGATCCTGGCTCAGAACGAACGCTGGCGGCAGGCTTAA<br>CACATGCAAGTCGAGCGCCCCGCAAGGGGAGCGGCAGACGGGTG<br>AGTAACGCGTGGGAATCTACCCTTGACTACGGAATAACGCAGGGA<br>AACTTGTGCTAATACCGTATGTGTCCTTCGGGAGAAAGATTTATC<br>GGTCAAGGATGAGCCCGCGTTGGATTAGCTAGTTGGTGGGGTAAA

	GGCCTACCAAGGCGACGATCCATAGCTGGTCTGAGAGGATGATCA<br>GCCACATTGGGACTGAGACACGGCCCAAACTCCTACGGGAGGCA<br>GCAGTGGGGAATATTGGACAATGGGCGCAAGCCTGATCCAGCCAT<br>GCCGCGTGAGTGATGAAGGCCCTAGGGTTGTAAAGCTCTTTCACC<br>GGAGAAGATAATGACGGTATCCGGAGAAGAAGCCCCGGCTAACT<br>TCGTGCCAGCAGCCGCGGTAATACGAAGGGGGCTAGCGTTGTTCG<br>GAATTACTGGGCGTAAAGCGCACGTAGGCGGATCGATCAGTCAG<br>GGGTGAAATCCCAGGGCTCAACCCTGGAACTGCCTTTGATACTGT<br>CGATCTGGAGTATGGAAGAGGTGAGTGGAATTCCGAGTGTAGAG<br>GTGAAATTCGTAGATATTCGGAGGAACACCAGTGGCGAAGGCGG<br>CTCACTGGTCCATTACTGACGCTGAGGTGCGAAAGCGTGGGGAGC<br>AAACAGGATTAGATACCCTGGTAGTCCACGCCGTAAACGATGAAT<br>GTTAGCCGTCGGGCAGTATACTGTTCGGTGGCGCAGCTAACGCAT<br>TAAACATTCCGCCTGGGGAGTACGGTCGCAAGATTAAAACTCAAA<br>GGAATTGACGGGGGCCCGCACAAGCGGTGGAGCATGTGGTTTAAT<br>TCGAAGCAACGCGCAGAACCTTACCAGCCCTTGACATGCCCGGCT<br>ACTTGCAGAGATGCAAGGTTCCCTTCGGGGACCGGGACACAGGTG<br>CTGCATGGCTGTCGTCAGCTCGTGTCGTGAGATGTTGGGTTAAGTC<br>CCGCAACGAGCGCAACCCTCGCCCTTAGTTGCCAGCATTCAGTTG<br>GGCACTCTAAGGGGACTGCCGGTGATAAGCCGAGAGGAAGGTGG<br>GGATGACGTCAAGTCCTCATGGCCCTTACGGGCTGGGCTACACAC<br>GTGCTACAATGGTGGTGACAGTGGGCAGCGAGCACGCGAGTGTG<br>AGCTAATCTCCAAAAGCCATCTCAGTTCGGATTGCACTCTGCAAC<br>TCGAGTGCATGAAGTTGGAATCGCTAGTAATCGCGGATCAGCATG<br>CCGCGGTGAATACGTTCCCGGGCCTTGTACACACCGCCCGTCACA<br>CCATGGGAGTTGGTTTTACCCGAAGGTAGTGCGCTAACCGCAAGG<br>AGGCAGCTAACCACGGTAGGGTCAGCGACTGGGGTGAAGTCGTA<br>ACAAGGTAGCCGTAGGGGAACCTGTGGCTGGATCACCTCCTTA
>MN067725.1	AGTTTGATCCTGGCTCAGAACGAACGCTGGCGGCAGGCTTAACAC<br>ATGCAAGTCGAGCGCCCCGCAAGGGGAGCGGCAGACGGGTGAGT<br>AACGCGTGGGAATCTACCCTTGACTACGGAATAACGTAGGGAAAC<br>TTGTGCTAATACCGTATGTGTCCTTCGGGAGAAAGATTTATCGGTC<br>AAGGATGAGCCCGCGTTGGATTAGCTAGTTGGTGGGGTAAAGGCC<br>TACCAAGGCGACGATCCATAGCTGGTCTGAGAGGATGATCAGCCA<br>CATTGGGACTGAGACACGGCCCAAACTCCTACGGGAGGCAGCAG<br>TGGGGAATATTGGACAATGGGCGCAAGCCTGATCCAGCCATGCCG<br>CGTGAGTGATGAAGGCCCTAGGGTTGTAAAGCTCTTTCACCGGAG<br>AAGATAATGACGGTATCCGGAGAAGAAGCCCCGGCTAACTTCGTG<br>CCAGCAGCCGCGGTAATACGAAGGGGGCTAGCGTTGTTCGGAATT<br>ACTGGGCGTAAAGCGCACGTAGGCGGATCGATCAGTCAGGGGTG<br>AAATCCCAGGGCTCAACCCTGGAACTGCCTTTGATACTGTCGATC<br>TGGAGTATGGAAGAGGTGAGTGGAATTCCGAGTGTAGAGGTGAA<br>ATTCGTAGATATTCGGAGGAACACCAGTGGCGAAGGCGGCTCACT<br>GGTCCATTACTGACGCTGAGGTGCGAAAGCGTGGGGAGCAAACA

	GGATTAGATACCCTGGTAGTCCACGCCGTAAACGATGAATGTTAG<br>CCGTCGGGCAGTATACTGTTCGGTGGCGCAGCTAACGCATTAAAC<br>ATTCCGCCTGGGGAGTACGGTCGCAAGATTAAAACTCAAAGGAAT<br>TGACGGGGGCCCGCACAAGCGGTGGAGCATGTGGTTTAATTCGAA<br>GCAACGCGCAGAACCTTACCAGCCCTTGACATGCCCGGCTACTTG<br>CAGAGATGCAAGGTTCCCTTCGGGGACCGGGACACAGGTGCTGCA<br>TGGCTGTCGTCAGCTCGTGTCGTGAGATGTTGGGTTAAGTCCCGC<br>AACGAGCGCAACCCTCGCCCTTAGTTGCCAGCATTCAGTTGGGCA<br>CTCTAAGGGGACTGCCGGTGATAAGCCGAGAGGAAGGTGGGGAT<br>GACGTCAAGTCCTCATGGCCCTTACGGGCTGGGCTACACACGTGC<br>TACAATGGTGGTGACAGTGGGCAGCGAGCACGCGAGTGTGAGCT<br>AATCTCCAAAAGCCATCTCAGTTCGGATTGCACTCTGCAACTCGA<br>GTGCATGAAGTTGGAATCGCTAGTAATCGCGGATCAGCATGCCGC<br>GGTGAATACGTTCCCGGGCCTTGTACACACCGCCCGTCACACCAT<br>GGGAGTTGGTTTTACCCGAAGGTAGTGCGCTAACCGCAAGGAGGC<br>AGCTAACCACGGTAGGGTCAGCGACTGGGGTGAAGTCGTAACAA<br>GGTAGCCGTAGGGGAACCTGCGGCTGGATCACCTCCTTAATC
>RP-8	GAATTCGTCGACAACAGAGTTTGATCCTGGCTCAGAACGAACGCT<br>GGCGGCAGGCTTAACACATGCAAGTCGAGCGCCCCGAAGGGGA<br>GCGGCAGACGGGTGAGTAACGCGTGGGAATCTACCCTTGACTACG<br>GAATAACGCAGGGAAACTTGTGCTAATACCGTATGTGTCCTTCGG<br>GAGAAAGATTTATCGGTCAAGGATGAGCCCGCGTTGGATTAGCTA<br>GTTGGTGGGGTAAAGGCCTACCAAGGCGACGATCCATAGCTGGTC<br>TGAGAGGATGATCAGCCACATTGGGACTGAGACACGGCCCAAAC<br>TCCTACGGGAGGCAGCAGTGGGGAATATTGGACAATGGGCGCAA<br>GCCTGATCCAGCCATGCCGCGTGAGTGATGAAGGCCCTAGGGTTG<br>TAAAGCTCTTTCACCGGAGAAGATAATGACGGTATCCGGAGAAGA<br>AGCCCCGGCTAACTTCGTGCCAGCAGCCGCGGTAATACGAAGGGG<br>GCTAGCGTTGTTCGGAATTACTGGGCGTAAAGCGCACGTAGGCGG<br>ATCGATCAGTCAGGTGTGAAATCCCAGGGCTCAACCCTGGAACTG<br>CCTTTGATACTGTCGATCTGGAGTATGGAAGAGGTGAGTGGAATT<br>CCGAGTGTAGAGGTGAAATTCGTAGATATTCGGAGGAACACCAGT<br>GGCGAAGGCGGCTCACTGGTCCATTACTGACGCTGAGGTGCGAAA<br>GCGTGGGGAGCAAACAGGATTAGATACCCTGGTAGTCCACGCCGT<br>AAACGATGAATGTTAGCCGTCGGGCAGTATACTGTTCGGTGGCGC<br>AGCTAACGCATTAAACATTCCGCCTGGGGAGTACGGTCGCAAGAT<br>TAAAACTCAAAGGAATTGACGGGGGCCCGCACAAGCGGTGGAGC<br>ATGTGGTTTAATTCGAAGCAACGCGCAGAACCTTACCAGCCCTTG<br>ACATGCCCGGCTACTTGCAGAGATGCAAGGTTCCCTTCGGGGACC<br>GGGACACAGGTGCTGCATGGCTGTCGTCAGCTCGTGTCGTGAGAT<br>GTTGGGTTAAGTCCCGCAACGAGCGCAACCCTCGCCCTTAGTTGC<br>CAGCATTCAGTTGGGCACTCTAAGGGGACTGCCGGTGATAAGCCG<br>AGAGGAAGGTGGGGATGACGTCAAGTCCTCATGGCCCTTACGGGC<br>TGGGCTACACACGTGCTACAATGGTGGTGACAGTGGGCAGCGAGC

	ACGCGAGTGTGAGCTAATCTCCAAAAGCCATCTCAGTTCGGATTG<br>CACTCTGCAACTCGAGTGCATGAAGTTGGAATCGCTAGTAATCGC<br>GGATCAGCATGCCGCGGTGAATACGTTCCCGGGCCTTGTACACAC<br>CGCCCGTCACACCATGGGAGTTGGTTTACCCGAAGGTAGTGCGC<br>TAACCGCAAGGAGGCAGCTAACCACGGTAGGGTCAGCGACTGGG<br>GTGAAGTCGTAACAAGGTAGCCGTAGGGGAACCTGCGGCTGGAT<br>CACCTCCTTAAGC
>T134	TGGCTCAGAACGAACGCTGGCGGCAGGCTTAACACATGCAAGTCG<br>AGCGCCCCGCAAGGGGAGCGGCAGACGGGTGAGTAACGCGTGGG<br>AATCTACCCTTGACTACGGAATAACGCAGGGAAACTTGTGCTAAT<br>ACCGTATGTGTCCTTCGGGAGAAAGATTTATCGGTCAAGGATGAG<br>CCCGCGTTGGATTAGCTAGTTGGTGGGGTAAAGGCCTACCAAGGC<br>GACGATCCATAGCTGGTCTGAGAGGATGATCAGCCACATTGGGAC<br>TGAGACACGGCCCAAACTCCTACGGGAGGCAGCAGTGGGGAATA<br>TTGGACAATGGGCGCAAGCCTGATCCAGCCATGCCGCGTGAGTGA<br>TGAAGGCCCTAGGGTTGTAAAGCTCTTTCACCGGAGAAGATAATG<br>ACGGTATCCGGAGAAGAAGCCCCGGCTAACTTCGTGCCAGCAGCC<br>GCGGTAATACGAAGGGGGCTAGCGTTGTTCGGAATTACTGGGCGT<br>AAAGCGCACGTAGGCGGATCGATCAGTCAGGGGTGAAATCCCAG<br>GGCTCAACCCTGGAACTGCCTTTGATACTGTCGATCTGGAGTATG<br>GAAGAGGTGAGTGGAATTCCGAGTGTAGAGGTGAAATTCGTAGA<br>TATTCGGAGGAACACCAGTGGCGAAGGCGGCTCACTGGTCCATTA<br>CTGACGCTGAGGTGCGAAAGCGTGGGGAGCAAACAGGATTAGAT<br>ACCCTGGTAGTCCACGCCGTAAACGATGAATGTTAGCCGTCGGGC<br>AGTATACTGTTCGGTGGCGCAGCTAACGCATTAAACATTCCGCCT<br>GGGGAGTACGGTCGCAAGATTAAAACTCAAAGGAATTGACGGGG<br>GCCCGCACAAGCGGTGGAGCATGTGGTTTAATTCGAAGCAACGCG<br>CAGAACCTTACCAGCCCTTGACATGCCCGGCTACTTGCAGAGATG<br>CAAGGTTCCCTTCGGGGACCGGGACACAGGTGCTGCATGGCTGTC<br>GTCAGCTCGTGTCGTGAGATGTTGGGTTAAGTCCCGCAACGAGCG<br>CAACCCTCGCCCTTAGTTGCCAGCATTCAGTTGGGCACTCTAAGG<br>GGACTGCCGGTGATAAGCCGAGAGGAAGGTGGGGATGACGTCAA<br>GTCCTCATGGCCCTTACGGGCTGGGCTACACACGTGCTACAATGG<br>TGGTGACAGTGGGCAGCGAGCACGCGAGTGTGAGCTAATCTCCAA<br>AAGCCATCTCAGTTCGGATTGCACTCTGCAACTCGAGTGCATGAA<br>GTTGGAATCGCTAGTAATCGCGGATCAGCATGCCGCGGTGAATAC<br>GTTCCCGGGCCTTGTACACACCGCCCGTCACACCATGGGAGTTGG<br>TTTTACCCGAAGGTAGTGCGCTAACCGCAAGGAGGCAGCTAACCA<br>CGGTAGGGTCAGCGACTGGGGTGAAGTCGTAACAAGGTAGCCGT<br>AGGGGAACCTGCGGCTGGATCACCTCCTTTCTAAGGAAGCTGTGG<br>AACTGGTAAGACGACCGGCTGGATCTTCGGATCTCCCCGGTATGA<br>ACCTTCCCGTGCTTTTTAGAACATAGATGGCACCAGTCAGGTGAC<br>CATCGAAACGTAATACGCCGCGTAGACTTCGGTCACGACGGTATG<br>GCGAGCTTTCGCCGTCCACGTTTCTCTTTCTTCAAGAAGACAAAAA

	ACCGTATCGACCGGTTCCCCGAATGGGCCCGTAGCTCAGTTGGTT<br>AGAGCACACGCTTGATAAGCGTGGGGTCGGAAGTTCAAGTCTTCC<br>CGGGCCCACCATTTGCTTGATGTGAATGATGGTTTGGGATGATGG<br>AATGGCAGTTGATGCGGATTGTTGCCGAACCCGGGATGTGTCCTG<br>GGCGATCGAGCTGATGGGGCTGTAGCTCAGCTGGGAGAGCACCTG<br>CTTTGCAAGCAGGGGGTCAGCGGTTCGATCCCGCTCAGCTCCACC<br>AAATCGATTGGTGTTGAGACTGACGGCATTTGTCTTTTGAAGAAA<br>TACAAGTTTTGCATCGGCTTTGAGCCTGATGCCTGTTCTGCATATA<br>TCGTGAAGAGAAGATTGATCTGGAGGCTTCCAGGTGTTTTGGGTT<br>CTGCCCAAAGCGTCCGAGCCCAGTTCCTGTGAACCCATGGATGGC<br>CTAGCCGGCCGGATGTGGTGGAGGGATTGGAGGTAGGAAGGAAG<br>CTTGTCGCTCTGGATCGTTGTTGTTTGCAGTCTTCGGACTGCATCT<br>GACGGACGATCGGATTACCGTTGCCTGACCGCGCGGTATCGGATC<br>CAATCTCGAGAAGCTGGTCTTAAGACAGGCTGCAAGTGGGCTGCT<br>CGGCGTAGCTCCAATAAAGCAGACCTGTCGAACACGTTAATGGCA<br>TTGTTGGATTGACTGGGTTGTAAAAGGTAACCCGGTCTGTTGCCGT<br>TTCTTTGAAGCGGGCAACGAGATGATGAGCATTGGCAATGAGAAC<br>GATTAAGTGTCGTAAGGGCATTTGGTGGATGCCTTGGCATGCACA<br>GGCGATGAAGGACGTGATACGCTGCGAAAAGCCGTGGGGAGCTG<br>CGAATGAGCTTTGATCC
>T111	TGGCTCAGAACGAACGCTGGCGGCAGGCTTAACACATGCAAGTCG<br>AGCGCCCCGCAAGGGGAGCGGCAGACGGGTGAGTAACGCGTGGG<br>AATCTACCCTTGACTACGGAATAACGCAGGGAAACTTGTGCTAAT<br>ACCGTATGTGTCCTTCGGGAGAAAGATTTATCGGTCAAGGATGAG<br>CCCGCGTTGGATTAGCTAGTTGGTGGGGTAAAGGCCTACCAAGGC<br>GACGATCCATAGCTGGTCTGAGAGGATGATCAGCCACATTGGGAC<br>TGAGACACGGCCCAAACTCCTACGGGAGGCAGCAGTGGGGAATA<br>TTGGACAATGGGCGCAAGCCTGATCCAGCCATGCCGCGTGAGTGA<br>TGAAGGCCCTAGGGTTGTAAAGCTCTTTCACCGGAGAAGATAATG<br>ACGGTATCCGGAGAAGAAGCCCCGGCTAACTTCGTGCCAGCAGCC<br>GCGGTAATACGAAGGGGGCTAGCGTTGTTCGGAATTACTGGGCGT<br>AAAGCGCACGTAGGCGGATCGATCAGTCAGGGGTGAAATCCCAG<br>GGCTCAACCCTGGAACTGCCTTTGATACTGTCGATCTGGAGTATG<br>GAAGAGGTGAGTGGAATTCCGAGTGTAGAGGTGAAATTCGTAGA<br>TATTCGGAGGAACACCAGTGGCGAAGGCGGCTCACTGGTCCATTA<br>CTGACGCTGAGGTGCGAAAGCGTGGGGAGCAAACAGGATTAGAT<br>ACCCTGGTAGTCCACGCCGTAAACGATGAATGTTAGCCGTCGGGC<br>AGTATACTGTTCGGTGGCGCAGCTAACGCATTAAACATTCCGCCT<br>GGGGAGTACGGTCGCAAGATTAAAACTCAAAGGAATTGACGGGG<br>GCCCGCACAAGCGGTGGAGCATGTGGTTTAATTCGAAGCAACGCG<br>CAGAACCTTACCAGCCCTTGACATGCCCGGCTACTTGCAGAGATG<br>CAAGGTTCCCTTCGGGGACCGGGACACAGGTGCTGCATGGCTGTC<br>GTCAGCTCGTGTCGTGAGATGTTGGGTTAAGTCCCGCAACGAGCG<br>CAACCCTCGCCCTTAGTTGCCAGCATTCAGTTGGGCACTCTAAGG

GGACTGCCGGTGATAAGCCGAGAGGAAGGTGGGGATGACGTCAA
GTCCTCATGGCCCTTACGGGCTGGGCTACACACGTGCTACAATGG
TGGTGACAGTGGGCAGCGAGCACGCGAGTGTGAGCTAATCTCCAA
AAGCCATCTCAGTTCGGATTGCACTCTGCAACTCGAGTGCATGAA
GTTGGAATCGCTAGTAATCGCGGATCAGCATGCCGCGGTGAATAC
GTTCCCGGGCCTTGTACACACCGCCCGTCACACCATGGGAGTTGG
TTTTACCCGAAGGTAGTGCGCTAACCGCAAGGAGGCAGCTAACCA
CGGTAGGGTCAGCGACTGGGGTGAAGTCGTAACAAGGTAGCCGT
AGGGGAACCTGCGGCTGGATCACCTCCTTTCTAAGGAAGCTGTGG
AACTGGTAAGACGACCGGCTGGATCTTCGGATCTCCCCGGTATGA
ACCTTCCCGTGCTTTTTAGAACATAGATGGCACCAGTCAGGTGAC
CATCGAAACGTAATACGCCGCGTAGACTTCGGTCACGACGGTATG
GCGAGCTTTCGCCGTCCACGTTTCTCTTTCTTCAAGAAGACAAAAA
ACCGTATCGACCGGTTCCCCGAATGGGCCCGTAGCTCAGTTGGTT
AGAGCACACGCTTGATAAGCGTGGGGTCGGAAGTTCAAGTCTTCC
CGGGCCCACCATTTGCTTGATGTGAATGATGGTTTGGGATGATGG
AATGGCAGTTGATGCGGATTGTTGCCGAACCCGGGATGTGTCCTG
GGCGATCGAGCTGATGGGGCTGTAGCTCAGCTGGGAGAGCACCTG
CTTTGCAAGCAGGGGGTCAGCGGTTCGATCCCGCTCAGCTCCACC
AAATCGATTGGTGTTGAGACTGACGGCATTTGTCTTTTGAAGAAA
TACAAGTTTTGCATCGGCTTTGAGCCTGATGCCTGTTCTGCATATA
TCGTGAAGAGAAGATTGATCTGGAGGCTTCCAGGTGTTTTGGGTT
CTGCCCAAAGCGTCCGAGCCCAGTTCCTGTGAACCCATGGATGGC
CTAGCCGGCCGGATGTGGTGGAGGGATTGGAGGTAGGAAGGAAG
CTTGTCGCTCTGGATCGTTGTTGTTTGCAGTCTTCGGACTGCATCT
GACGGACGATCGGATTACCGTTGCCTGACCGCGCGGTATCGGATC
CAATCTCGAGAAGCTGGTCTTAAGACAGGCTGCAAGTGGGCTGCT
CGGCGTAGCTCCAATAAAGCAGACCTGTCGAACACGTTAATGGCA
TTGTTGGATTGACTGGGTTGTAAAAGGTAACCCGGTCTGTTGCCGT
TTCTTTGAAGCGGGCAACGAGATGATGAGCATTGGCAATGAGAAC
GATTAAGTGTCGTAAGGGCATTTGGTGGATGCCTTGGCATGCACA
GGCGATGAAGGACGTGATACGCTGCGAAAAGCCGTGGGGAGCTG
CGAATGAGCTTTGATCCATGGATCT
